# Longitudinal symptom severity tracking in vagus nerve stimulation patients: a 2-stage LLM-based pipeline with explainable AI

**DOI:** 10.1093/jamiaopen/ooag195

**Published:** 2026-10-01

**Authors:** Vedansh Thakkar, Greg M Silverman, Maura Putzer, Abhinab Kc, Sana Ikramuddin, Tanya Melnik, Nicholas E Ingraham, Nathan Keller, Samantha King, Emma Jones, Angela Bailey, Genevieve B Melton, Christopher Tignanelli

**Affiliations:** Department of Surgery, University of Minnesota, Minneapolis, MN 55455, United States; Natural Language Processing/Information Extraction Program, University of Minnesota, Minneapolis, MN 55455, United States; Department of Surgery, University of Minnesota, Minneapolis, MN 55455, United States; Natural Language Processing/Information Extraction Program, University of Minnesota, Minneapolis, MN 55455, United States; Department of Surgery, University of Minnesota, Minneapolis, MN 55455, United States; University of Minnesota Medical School, Minneapolis, MN 55455, United States; University of Minnesota Medical School, Minneapolis, MN 55455, United States; Department of Surgery, University of Minnesota, Minneapolis, MN 55455, United States; Natural Language Processing/Information Extraction Program, University of Minnesota, Minneapolis, MN 55455, United States; Department of Pulmonary, Allergy, Critical Care, and Sleep Medicine, University of Minnesota, Minneapolis, MN 55455, United States; Center for Learning Health Systems Sciences, University of Minnesota, Minneapolis, MN 55455, United States; University of Minnesota Medical School, Minneapolis, MN 55455, United States; Department of Surgery, University of Washington, Seattle, WA 98195, United States; Department of Surgery, University of Minnesota, Minneapolis, MN 55455, United States; University of Minnesota Medical School, Minneapolis, MN 55455, United States; Department of Surgery, University of Minnesota, Minneapolis, MN 55455, United States; Natural Language Processing/Information Extraction Program, University of Minnesota, Minneapolis, MN 55455, United States; Center for Learning Health Systems Sciences, University of Minnesota, Minneapolis, MN 55455, United States; Department of Surgery, University of Minnesota, Minneapolis, MN 55455, United States; Natural Language Processing/Information Extraction Program, University of Minnesota, Minneapolis, MN 55455, United States; Center for Learning Health Systems Sciences, University of Minnesota, Minneapolis, MN 55455, United States

**Keywords:** vagus nerve stimulation, large language models, symptom severity, longitudinal analysis, explainable AI, health informatics

## Abstract

**Objectives:**

To produce the first structured longitudinal dataset of symptom severity trajectories in vagus nerve stimulation (VNS) patients using a 2-stage large language model pipeline for automated symptom severity extraction from clinical notes, as part of the NIH-funded research evaluating vagal excitations and anatomical linkages (U54AT012307).

**Materials and methods:**

We analyzed 1427 annotated clinical notes from 95 patients across 5 corpora. LLaMA models (3.2-1B, 3.2-3B, and 3.3-70B) were fine-tuned to classify 13 symptoms as present, absent, or negated (stage 1), then assessed severity (indeterminate, mild, moderate, or severe; stage 2). Longitudinal trajectories were tracked quarterly in 23 VNS patients against a 10-patient surgical control cohort (NSQIP).

**Results:**

Severity F1-scores ranged from 0.76 to 0.96, and symptom classification reached 0.99 for high-prevalence symptoms, with the validation corpus (VNS vs NSQIP) as the dominant factor (29.4% of variance; no significant source-corpus effect), supporting cross-domain generalizability. Macro F1-scores were lower than weighted F1-scores for smaller models and NSQIP; suicidal-ideation sensitivity was lower on NSQIP than VNS (0.80 vs 1.00, largest model), limiting generalization for psychiatric risk detection. Equity analyses were exploratory given small subgroups (gender precision 0.95). The longitudinal dataset revealed a median time to clinically meaningful seizure improvement of 0.5 quarters, with 50% responders, 25% non-responders, and 25% worsened. Post-surgical decline was absent in the unmatched NSQIP control cohort, consistent with, but not confirming, VNS specificity.

**Discussion:**

To our knowledge, this is the first system producing structured symptom trajectories from free-text VNS documentation, supporting individualized over population-level monitoring.

**Conclusion:**

This dataset supports future treatment-response prediction, personalized device programming, and a reproducible clinical natural langauge processing benchmark for neuromodulation research.

## Background and significance

Vagus nerve stimulation (VNS) has emerged as a significant therapeutic intervention for various neurological and psychiatric conditions, particularly in the management of drug-resistant epilepsy, offering an alternative treatment option for patients who have not responded to traditional therapies.^1^ For example, VNS has been shown to reduce the frequency and severity of seizures in some patients with epilepsy.^2^ Recent research indicates that VNS is also highly effective for long-term treatment, reducing treatment-resistant depression severity by nearly 60% on average and achieving an 87% response rate.^3^

In clinical trials, research studies often incorporate extensive structured data, including laboratory results, vital signs, procedures, and diagnoses. However, a critical missing component is the characterization of patient symptoms and their severity. Traditionally, clinical trials have relied on specially trained nurses to assess patient symptoms or have used patient surveys to capture symptom presence and severity.^4^

Previous studies such as those by Silverman et al^5^ compared 2 natural language processing (NLP) pipelines for identifying active COVID-19 based on the Center for Disease Control’s COVID-19 symptom list. These approaches have been broadly successful; nevertheless, they continue to rely on a fixed set of rules.

Exponential advances in large language models (LLMs) offer a promising solution to the limitations of traditional symptom-tracking methods. Unlike rule-based systems, LLMs can dynamically extract symptoms and severity from unstructured clinical notes, critical for VNS patients where longitudinal symptom data guides therapy optimization. This work is conducted as part of the NIH-funded Research Evaluating Vagal Excitation and Anatomical Linkages (REVEAL) center (U54AT012307), a multi-institutional initiative across 8 academic medical centers with the primary aim of producing foundational datasets on multi-organ effects of VNS. To fulfill this aim, we develop and validate a 2-stage LLM pipeline fine-tuning LLaMA models (3.2-1B, 3.2-3B, and 3.3-70B)^6^ for symptom classification and applying prompt engineering for severity assessment, enabling the first structured longitudinal symptom severity dataset for VNS patients derived directly from real-world clinical notes rather than periodic surveys or standardized rating scales.

## Methods

### Study design and data sources

We obtained 1427 clinical notes from 95 patients across 5 corpora: (1) M Health Fairview (MHFV)-COVID: emergency department (ED) visit notes for acute COVID-19 patients; (2) MHFV-Post Acute Sequelae of SARS-CoV-2 (PASC): ED and outpatient notes with longitudinal follow-up for persistent post-COVID symptoms; (3) National COVID Cohort Collaborative (N3C)-COVID: de-identified notes from Mayo Clinic for acute COVID-19 patients; (4) National Surgical Quality Improvement Program (NSQIP): a surgical outcomes database of major procedures in adult patients; and (5) MHFV-VNS: ED visit notes for patients who underwent VNS procedures at MHFV.

Inclusion criteria for MHFV-COVID and MHFV-PASC are described in Silverman et al.^7^ MHFV-PASC additionally required no baseline symptoms, presentation >30 days post-infection, and at least 1 new or residual symptom. N3C-COVID notes were restricted to within 2 weeks before and 4 weeks after the first positive COVID-19 result, containing >1000 characters, with up to 20 notes per patient.^8^ MHFV-VNS included inpatients aged ≥18 presenting at MHFV between January 2011 and June 2023 who consented to research use.

MHFV-COVID, MHFV-PASC, and N3C-COVID notes were manually annotated for symptom presence, absence, and negation by 3 subject matter experts (SMEs) (inter-rater reliability [IRR] = 0.68, Fleiss’ kappa).^9^ NSQIP and VNS notes were annotated for symptom severities by 5 SMEs (IRR = 0.72 for symptoms, 0.60 for severity).

All corpora were partitioned at the patient level: every note from a given patient was assigned exclusively to the training, validation, or test set, precluding cross-partition patient overlap. The 3 COVID-era corpora were assigned entirely to training, and the MHFV-VNS and NSQIP corpora were each split across all 3 partitions.

### NSQIP as a surgical control cohort

The NSQIP corpus served a dual role. Primarily, NSQIP patients functioned as a surgical control cohort: patients who underwent major surgery without VNS implantation, enabling a case-control comparison of longitudinal symptom severity trajectories with the hypothesis that VNS-relevant symptoms would not show a decreasing severity trend in the absence of neuromodulatory intervention. Secondarily, NSQIP served as a domain-generalization test set to evaluate whether the fine-tuned (FT) LLM generalizes to non-VNS surgical populations.

### Corpora performance effects and symptom distribution

To evaluate how corpus characteristics influenced classification performance, we conducted an analysis of variance (ANOVA) on per-symptom weighted F1-scores with 3 predictors: corpus source (the 5 source datasets: MHFV-COVID, MHFV-PASC, N3C-COVID, NSQIP, and MHFV-VNS), the validation corpus used for early stopping during training (a binary factor with levels VNS and NSQIP; coded as validation_group in Tables S3, S4, and S6 in Appendix SA), and log-transformed mean symptom-specific TF-IDF, together with the corpus-by-TF-IDF interaction (TF-IDF computation detailed in Appendix SB). Effect sizes (η²) were calculated throughout to quantify the magnitude of observed differences.

### Annotation schema

Annotators identified symptoms and severities within the history of present illness and other relevant note sections across 3 iterative rounds of 20 overlapping notes each. Severity labels followed a 6-point integer scale: −2 (not mentioned), −1 (negated), 0 (present, severity not inferable), 1 (mild), 2 (moderate), 3 (severe). After each round, IRR scores were computed, disagreement patterns identified, and guidelines refined to reduce annotator divergence.

### LLM fine-tuning for symptom classification

We employed a 2-stage approach for symptom classification and severity assessment. In stage 1, LLaMA models (3.2-1B, 3.2-3B, and 3.3-70B) were FT to classify each of 13 key VNS symptoms (identified by prevalence analysis) by status as present, negated, or absent. To avoid a second annotation pass, stage 1 status labels were derived directly from the annotation scale: not mentioned (−2) was mapped to absent, negated (−1) was retained as negated, and any present symptom (severity 0 through 3) was mapped to present. In stage 1 we trained only the final classification layer of each LLaMA model while keeping all pretrained weights frozen; low-rank adapters were not used. Each model was configured as 13 parallel 3-class classifiers, yielding a 39-neuron output layer (13 symptoms × 3 status labels). Full fine-tuning details with hyperparameters are provided in Appendix SB (Table S15).

### LLM prompting for severity assessment

In the second stage, severity assessment was performed using prompt engineering.^10^ The best-performing model from the symptom classification stage (LLaMA-3.3-70B) was prompted to assess severity as indeterminate, mild, moderate, or severe, focusing exclusively on clinical note summaries, since symptom classification achieved higher F1-scores with summarized notes than full notes. A direct comparison of summarized vs full notes for classification is reported in Appendix SB (Table S16) and motivated this choice; an analogous severity-specific full-note comparison was not performed and is a direction for future work. Summaries were generated by LLaMA3-OpenBioLLM-70B^11^ using prompts developed with clinical SMEs. To verify that summarization did not drop clinically relevant content, 50 summaries were audited by an attending physician against their source notes, with no clinically relevant omissions identified. Additional details of summary notes vs full notes, severity assessment, output post-processing, and response failure management are provided in Appendix SB.

### FT vs off-the-shelf LLM comparison

To contextualize the value of fine-tuning, the off-the-shelf LLaMA-3.3-70B model was evaluated on the same test set using the same input format as the FT variants, replicating the experimental design of Thakkar et al.^12^ Performance was assessed using weighted F1-score and structured output consistency, defined as the proportion of responses conforming to the expected JSON format without manual correction.

### Longitudinal symptom trajectory modeling and LLM validation

We modeled longitudinal symptom severity trajectories from SME-annotated clinical notes across 23 VNS patients, comparing SME ground truth against LLM predictions for 13 symptoms using the ordinal burden scale defined above. All longitudinal quantities were defined per patient and per symptom on this scale. A documented quarter was any quarter with at least 1 note mentioning the symptom, with multiple notes averaged to that quarter’s mean; quarters coded -2 (not mentioned) were treated as missing and excluded. Baseline was the mean severity across pre-surgical documented quarters in which the symptom was present (codes 0 to 3); pre-surgical negated quarters (−1) were excluded so that baseline reflects symptomatic burden. Post-surgical quarterly means included negated quarters (−1), which denote explicit symptom absence and therefore genuine improvement. Relative to baseline, we defined 3 nested quantities. A first observed reduction was the earliest post-surgical documented quarter with mean severity at least 1 level below baseline. A clinically meaningful improvement was a reduction of at least 1 level below baseline maintained across 2 consecutive documented post-surgical quarters. A sustained improvement was a clinically meaningful improvement that persisted, with severity remaining at least 1 level below baseline through the final documented post-surgical quarter. Because clinical documentation in this cohort was sparse and irregular, consecutiveness was defined over documented quarters without a maximum-gap constraint: 2 successive documented post-surgical quarters were treated as consecutive regardless of the number of intervening quarters with no symptom mention. Requiring adjacent calendar quarters would otherwise exclude most patients from any improvement or worsening determination. Time to improvement, reported throughout as the primary metric, was the number of quarters from surgery to the first quarter of a clinically meaningful improvement. Patients were then classified by terminal trajectory, based on whether the final documented post-surgical quarter was at least 1 severity level above baseline. Patients ending above baseline were classified as worsened, regardless of earlier transient improvement; patients with a clinically meaningful improvement not ending above baseline were responders; all others were non-responders. This terminal-trajectory rule prevents patients who improve and later relapse from being counted as responders, which matters in this sparsely documented cohort where relapses may not form 2 consecutive worsening quarters. Cohort-level analysis required a minimum of 5 clinical notes in both pre- and post-surgical periods.

### SHAP-based explainable AI (XAI): inter-symptom severity modeling and explainability

Beyond per-note severity assessment, we examined inter-symptom structure in the pipeline’s outputs by training a downstream OrdinalGBT model^13^ on the per-symptom severity predictions and interpreting it with SHAP (SHapley Additive exPlanations)^14^. This analysis explains the OrdinalGBT model’s inter-symptom predictions, not the LLM’s internal decisions. Detailed model configuration is provided in Appendix SB.

### Statistical analysis

Model performance was evaluated using weighted precision, recall, and F1-score on the test set for both symptom classification (present, absent, and negated) and severity assessment (mild, moderate, and severe), accounting for class imbalance. Because weighted metrics can be dominated by the majority class, we additionally computed macro-averaged precision, recall, and F1-score, along with per-class metrics and confusion matrices, for symptom classification. Equity analyses evaluated performance across racial and gender subgroups. Bootstrapped 95% CIs were estimated with 1000 resamples, and paired *t*-tests on bootstrapped distributions assessed statistical significance of performance differences across model sizes and input modalities (full vs summarized notes, Appendix SB), with *P* < .05 considered significant.

## Results

### Dataset partitioning for LLM fine-tuning

A total of 1427 clinical notes from 95 patients were partitioned at the patient level into training, validation, and testing sets (Table 1). All notes from a given patient were assigned exclusively to a single partition, so that no patient contributed notes to more than 1 set. Training comprised 58 unique patients (857 notes; 60%), validation 8 patients (103 notes; 7%), and testing 29 patients (467 notes; 33%). This strategy assigned the larger COVID-related corpora entirely to training while reserving the majority of VNS and NSQIP notes for testing, enabling evaluation of cross-domain generalization from predominantly COVID-trained data to VNS and surgical contexts. The held-out test set contained 23 MHFV-VNS and 6 NSQIP patients.

**Table 1.** Demographic characteristics of study population, including patient and note counts by corpus and partition.

Corpus	Number of patients/notes	Median age in years, IQR (Q1, Q3)	Male %	Racial distribution & ethnicity %
**MHFV-COVID**		54.68 (39.00, 66.64)	54.00 %	9.09 % Asian 27.27 % Black 4.54 % Hispanic 7.10 % others* 52.00 % White
** - Training**	3/46			
** - Validation**	0/0			
** - Testing**	0/0			
**MHFV-PASC**		53.10 (38.00, 67.90)	58.00 %	5.00 % Asian 11.90 % Black 3.36 % Hispanic 15.74 % others* 64.00 % White
** - Training**	32/476			
** - Validation**	0/0			
** - Testing**	0/0			
N3C-COVID**		Not available (N/A)	N/A	N/A
** - Training**	10/148			
** - Validation**	0/0			
** - Testing**	0/0			
**NSQIP**		54.78 (40.90, 67.09)	32.00%	0.66 % Asian 4.00 % Black 1.33 % Hispanic 6.66 % others* 87.33% White
** - Training**	2/23			
** - Validation**	2/22			
** - Testing**	6/105			
**MHFV-VNS**		52.07 (35.80, 62.89)	54.25%	4.00% Asian 9.34% Black 86.66% White
** - Training**	11/164			
** - Validation**	6/81			
** - Testing**	23/362			

All corpora were partitioned at the patient level; no patient contributed notes to more than 1 partition.

* Others include declined races.

** N3C-COVID dataset is de-identified. IQR: interquartile range.

### Corpus prevalence analysis

Symptom prevalence varied substantially across corpora (Table S1 in Appendix SA). Pain was most prevalent in N3C COVID (0.300) and NSQIP (0.311); nausea and vomiting in MHFV-COVID (0.309); and seizure in VNS (0.273), well above other corpora (0.017-0.030). Within VNS, moderate-to-severe profiles were most frequent for depression and seizure, followed by pain. Symptom distributions across splits and severity annotations are presented in Tables S2-S3, Appendix SA.

### Corpora performance effects analysis

ANOVA identified 2 significant predictors of symptom classification F1-scores (*P* < .001): log-transformed TF-IDF values and the validation corpus used during training (VNS vs NSQIP; Table S4 in Appendix SA). Corpus membership alone was not significant (*P* > .05), but its interaction with log TF-IDF was (*P* < .01). For severity classification (Table S5 in Appendix SA), the validation corpus was again significant (*P* < .01); log TF-IDF and corpus membership alone were not, but their interaction remained significant (*P* < .01), suggesting term-frequency effects on severity varied by corpus. This is consistent with the keyword analysis (Table S6 in Appendix SA): corpora with richer symptom-specific vocabulary (eg, VNS) showed stronger TF-IDF effects than sparser corpora (eg, COVID). η^2^ analysis confirmed that the validation corpus accounted for 29.4% of variance in symptom classification, while 92.0% of variance in severity classification remained unexplained (Table S7 in Appendix SA).

### Symptom classification performance

Figure 1 (Table S8 in Appendix SA) presents weighted precision, recall, and F1-scores for symptom classification across FT LLaMA models on the VNS and NSQIP test sets. LLaMA-3.3-70B outperformed all other models, reaching F1-scores of 0.98 for seizures and 0.99 for self-harm, with lower performance for subjective symptoms (pain 0.63 for LLaMA-3.2-1B). Performance was consistently higher on VNS than NSQIP notes (seizure F1 0.98 vs 0.91). Because weighted F1 can be dominated by the majority absent class, we also computed macro-averaged precision, recall, F1, per-class metrics, and confusion matrices (Table S9 in Appendix SA; Table S17 in Appendix SC; Table S18 in Appendix SD). Macro F1-scores were consistently lower than weighted F1-scores, particularly for smaller models and NSQIP, reflecting reduced performance on the minority present and negated classes.

**Figure 1.**
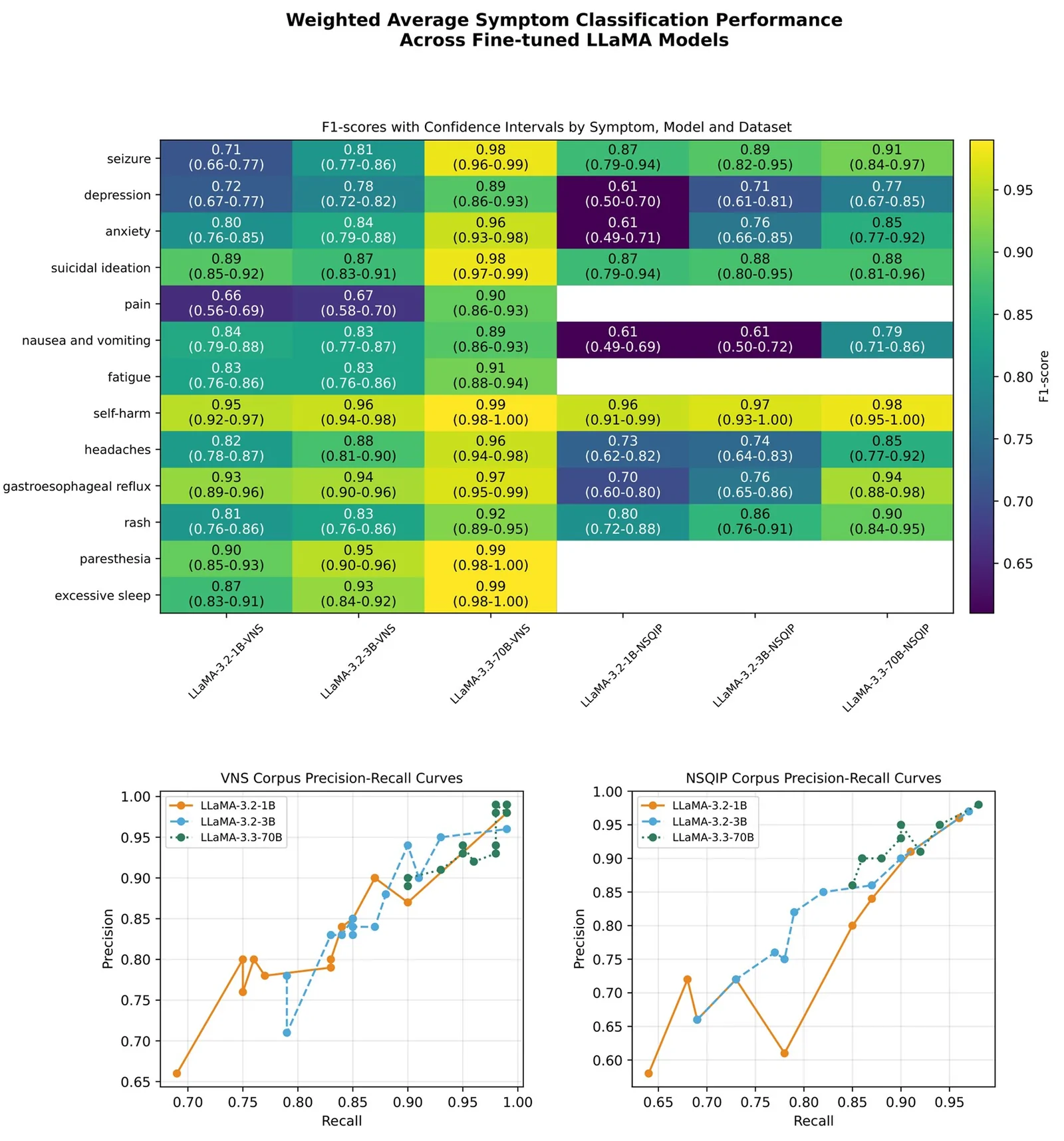
Weighted average symptom classification performance across fine-tuned LLaMA models.

Given their clinical importance, we separately report sensitivity and false-negative rate for suicidal ideation and self-harm (Table S10 in Appendix SA). Suicidal-ideation sensitivity on VNS was high for the larger models (1.00 for LLaMA-3.3-70B, 0.98 for LLaMA-3.2-3B) but low for LLaMA-3.2-1B (0.30); on NSQIP, sensitivity was lower for all models, following the same model-size ordering (0.80, 0.70, and 0.50). Self-harm sensitivity ranged from 0.50 to 1.00 but rests on very few present cases (2 to 4 per cohort) and should be read with corresponding caution.

### Severity assessment performance

Figure 2 (Table S11 in Appendix SA) presents severity assessment for LLaMA-3.3-70B on the VNS and NSQIP test sets. Severity F1-scores ranged from 0.76 to 0.96 (seizures 0.85, self-harm 0.89), with lower performance for subjective symptoms (anxiety 0.84, pain 0.80). Performance was higher on VNS than NSQIP notes (nausea and vomiting 0.84 vs 0.73).

**Figure 2.**
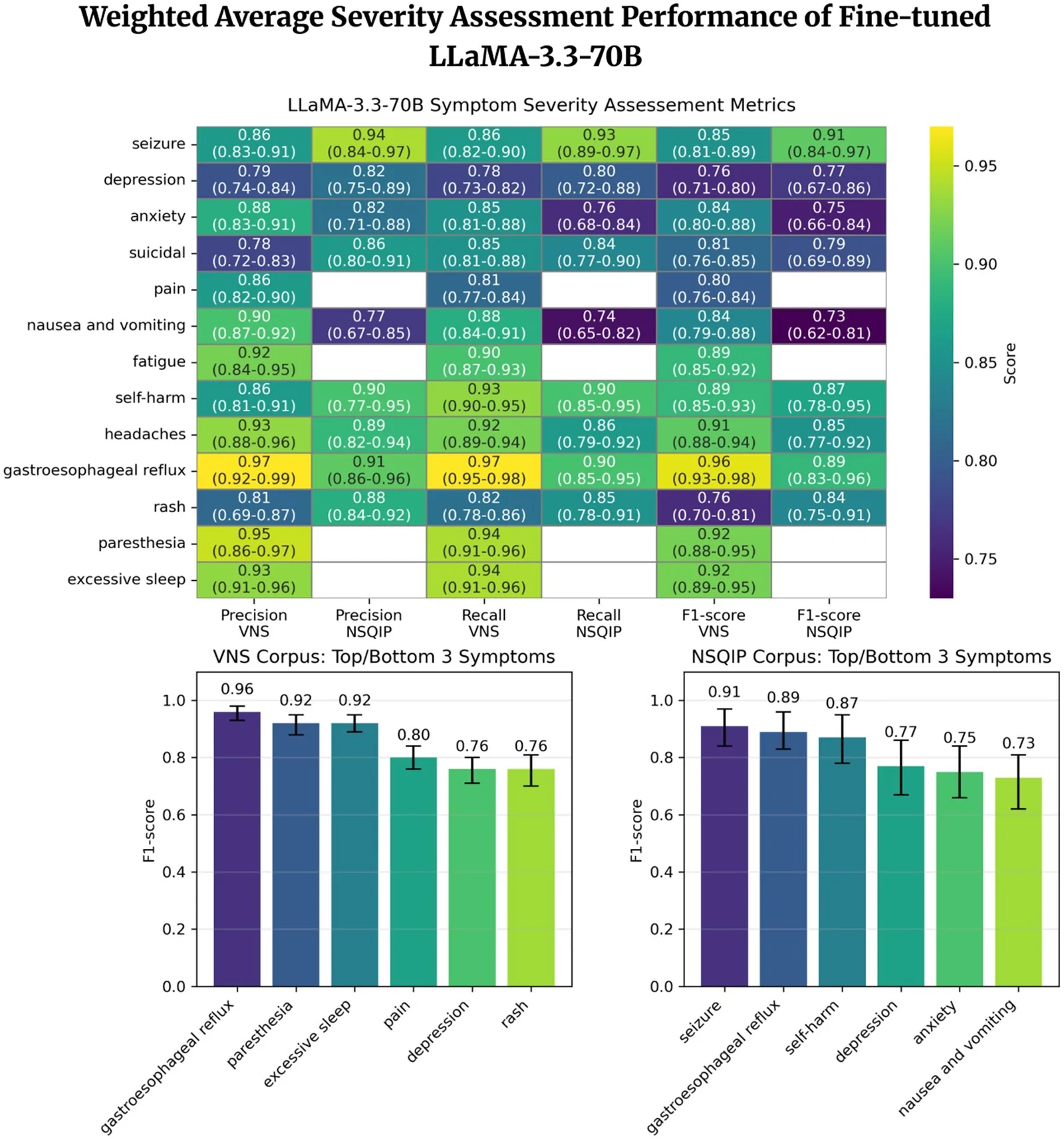
Weighted average severity assessment performance of fine-tuned LLaMA-3.3-70B.

### FT vs off-the-shelf LLaMA-3.3-70B

Consistent with Thakkar et al^12^ fine-tuning substantially improved performance and output formatting over the off-the-shelf LLaMA-3.3-70B: weighted F1 rose from 0.75 to 0.92 and JSON output consistency from 80% to 100%. Gains were largest for low-prevalence symptoms (gastroesophageal reflux, rash, and paresthesia), where un-fine-tuned (UFT) models defaulted to absent. Per-symptom comparisons are presented in Table 2.

**Table 2.** Per-symptom weighted F1-scores with 95% confidence intervals for FT and off-the-shelf UFT LLaMA-3.3-70B on the VNS test set.

Symptom	FT F1-Score (95% CI)	UFT F1-Score (95% CI)	Absolute gain (FT—UFT)
**Seizure**	0.98 (0.96-0.99)	0.84 (0.81-0.87)	+0.14
**Depression**	0.91 (0.88-0.94)	0.76 (0.71-0.81)	+0.15
**Anxiety**	0.89 (0.86-0.92)	0.75 (0.70-0.80)	+0.14
**Suicidal ideation**	0.94 (0.91-0.96)	0.80 (0.75-0.85)	+0.14
**Pain**	0.88 (0.84-0.92)	0.73 (0.67-0.79)	+0.15
**Nausea and vomiting**	0.91 (0.87-0.94)	0.76 (0.70-0.82)	+0.15
**Fatigue**	0.93 (0.91-0.95)	0.81 (0.77-0.85)	+0.12
**Self-harm**	0.99 (0.98-1.00)	0.88 (0.84-0.92)	+0.11
**Headaches**	0.90 (0.87-0.93)	0.77 (0.72-0.82)	+0.13
**Gastroesophageal reflux**	0.96 (0.91-1.00)	0.63 (0.54-0.72)	+0.33
**Rash**	0.88 (0.82-0.94)	0.58 (0.49-0.67)	+0.30
**Paresthesia**	0.87 (0.81-0.93)	0.65 (0.57-0.73)	+0.22
**Excessive sleep**	0.92 (0.89-0.95)	0.78 (0.73-0.83)	+0.14

### Equity analysis

Figure 3 (Table S12 in Appendix SA) presents equity analyses across racial and gender subgroups for VNS and NSQIP test sets. LLaMA-3.3-70B showed consistent performance across gender subgroups (male precision 0.95; 95% CI, 0.92-0.97; female 0.95; 95% CI, 0.91-0.97) and racial groups, while smaller models showed greater demographic sensitivity (eg, LLaMA-3.2-3B F1: 0.83 for Asian vs 0.89 for Black patients). In the NSQIP dataset, gender equity was similarly consistent, whereas racial subgroups were very small (1 Asian and 2 Black participants). These equity analyses are exploratory: several demographic subgroups contained very few patients, which limits statistical power and precludes strong conclusions about model fairness. Per-subgroup patient and note counts are reported in Table S13 in Appendix SA.

**Figure 3.**
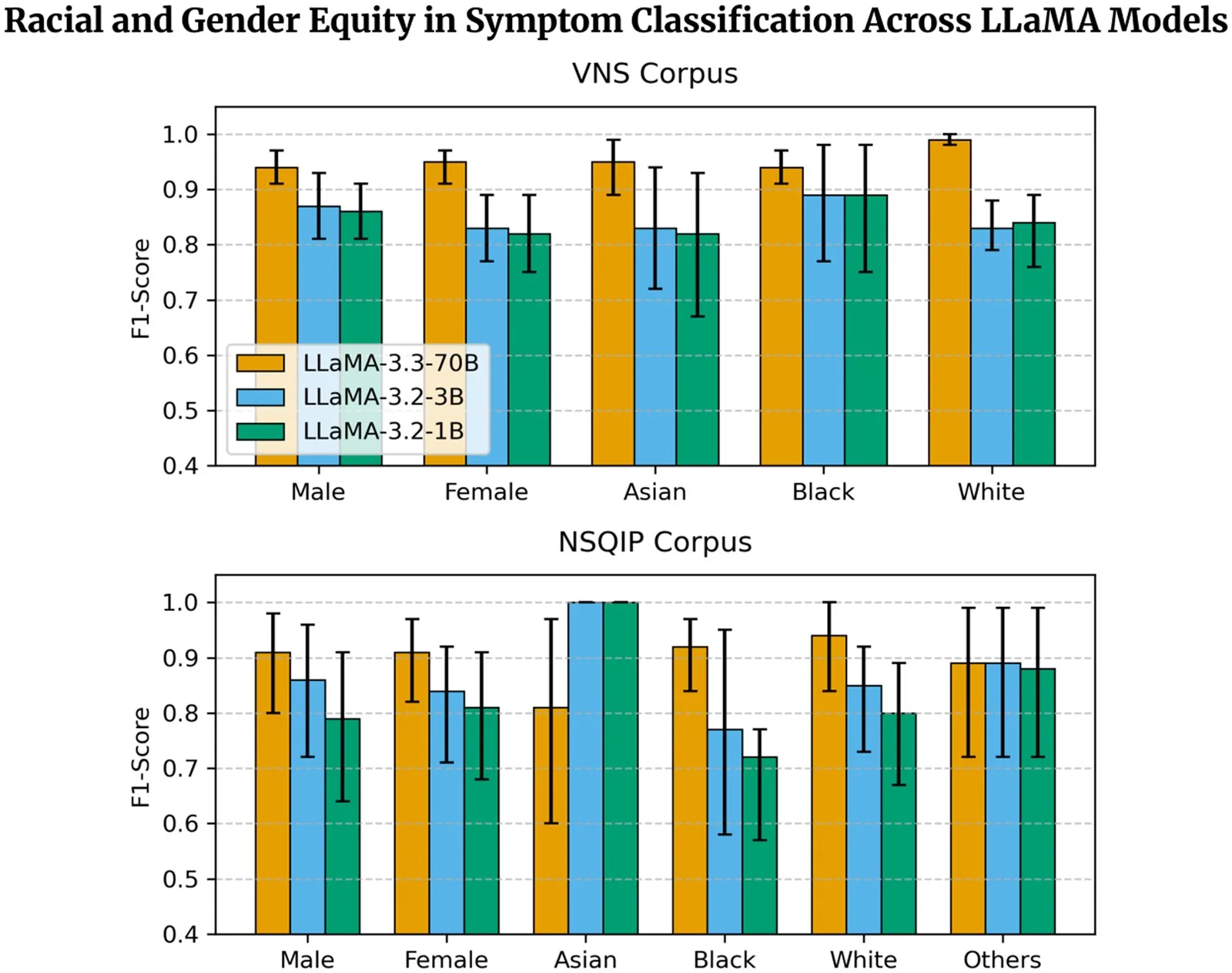
Racial and gender equity in symptom classification across LLaMA model sizes.

### Longitudinal symptom trajectories and LLM validation results

The longitudinal analysis tracked symptom severity across pre- and post-surgical notes for 23 VNS patients using both SME ground-truth labels and LLM predictions.

#### Individual patient trajectories

At the individual level, Figure 4 illustrates a representative VNS patient whose mean seizure severity fell from 1.80 at baseline to 0.00 post-surgically, a first observed reduction within the first quarter followed by clinically meaningful improvement. Because a score of 0 denotes a present but ungradable symptom rather than absence, this is an absolute reduction of 1.80 points, not a percentage reduction. SME and LLM trajectories aligned closely throughout, including in additional patients shown in Figure S1  Appendix SA.

**Figure 4.**
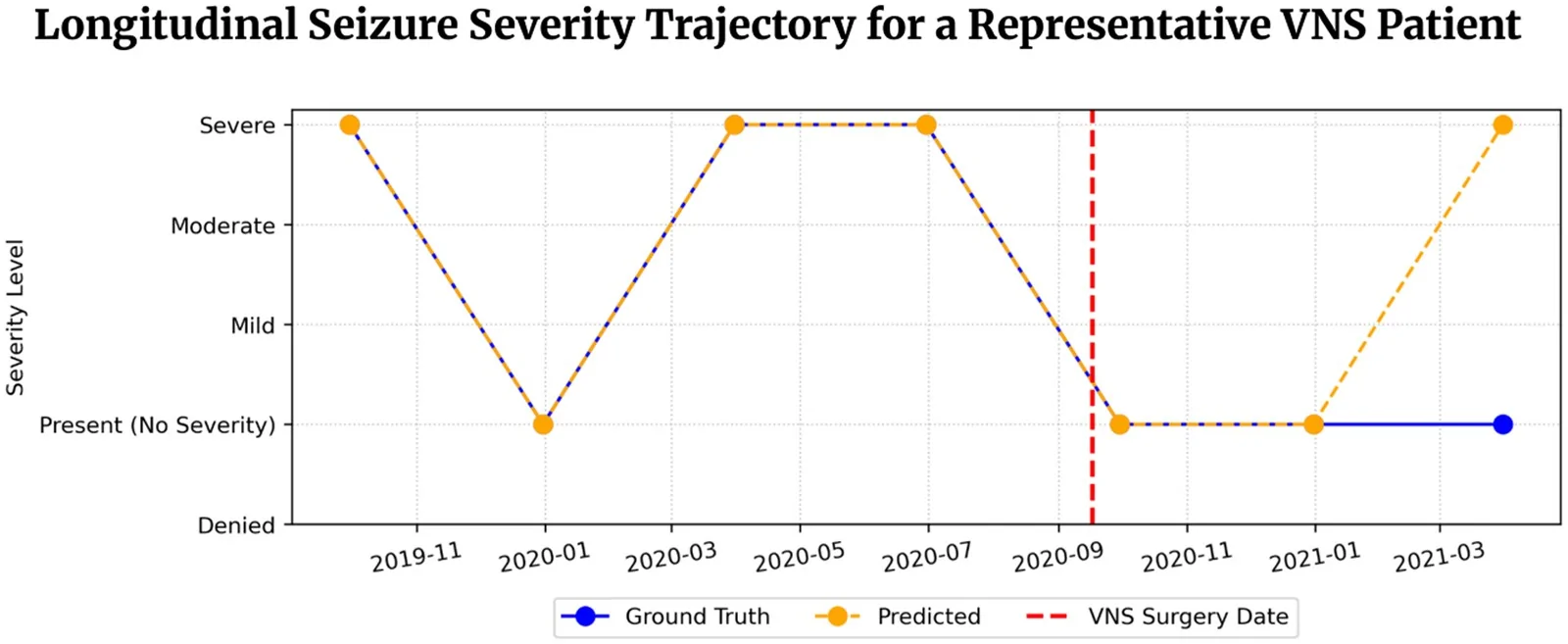
Longitudinal seizure severity trajectory for a representative VNS patient.

#### Cohort level seizure analysis

Cohort-level analysis was restricted to 8 patients meeting the minimum documentation threshold (5 clinical notes in both periods; Figure 5). The heatmap reveals substantial heterogeneity: P16 is the clearest responder, with severe pre-surgical seizures (severity 3.0) followed by sustained reduction to the lowest documented levels, consistent with Figure 4; P2 shows a similar reduction. Of the 4 responders, P1 and P16 additionally met the stricter sustained-improvement criterion (at least 1 level below baseline through their final quarter), while P2 and P8 drifted back toward baseline. P13 recurred at quarters 15, 18, 19, and 23, and P3 recurred late at quarter 34. P11 and P23 had sparse seizure mentions and neither met the improvement criterion, though sparse documentation warrants caution. Pre- vs post-surgical changes are quantified in Figure S2  Appendix SA; responder classifications are presented in Table 3.

**Figure 5.**
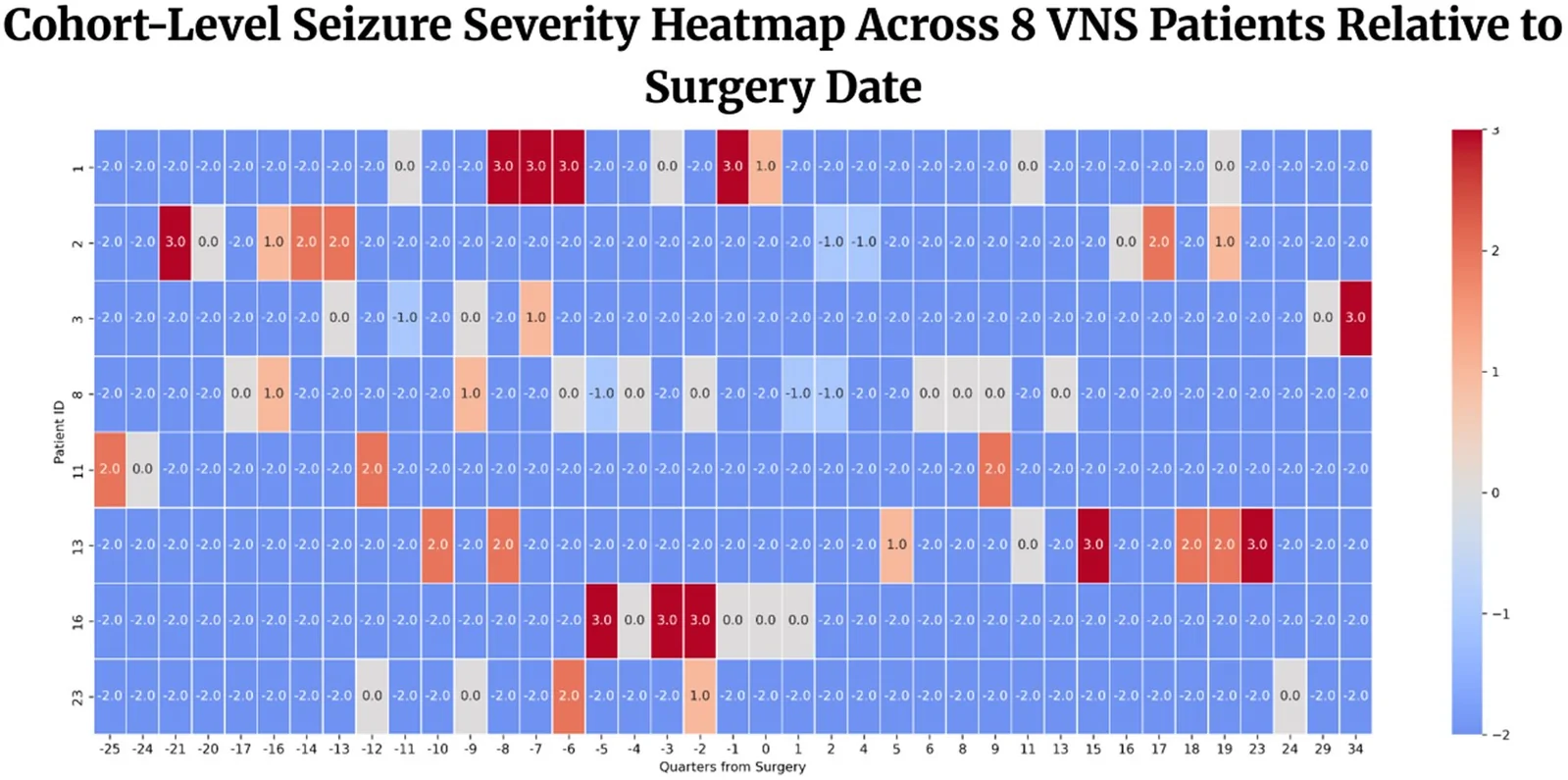
Cohort-level seizure severity heatmap across 8 VNS patients relative to surgery date.

**Table 3.** Responder classification of VNS patients based on longitudinal seizure severity trajectories (*n* = 8).

Patient ID	Pre-VNS mean severity	Post-VNS mean severity	Change in severity	Responder classification	Time to improvement (quarters)
**P1**	2.00	0.33	−1.67	Responder	0
**P2**	1.60	0.20	−1.40	Responder	2
**P3**	0.33	1.50	+1.17	Worsened	N/A
**P8**	0.33	−0.33	−0.66	Responder	1
**P11**	1.33	2.00	+0.67	Non-responder	N/A
**P13**	2.00	1.83	−0.17	Worsened	N/A
**P16**	1.80	0.00	−1.80	Responder	0
**P23**	0.75	0.00	−0.75	Non-responder	N/A

Pre- and post-surgical mean severity scores are computed from SME-annotated clinical notes, excluding quarters with no symptom mention (coded as −2). The surgery quarter (quarter 0) is treated as post-surgical; only notes dated after the procedure contribute to it. Change in severity is calculated as post-surgical mean minus pre-surgical mean, where negative values indicate improvement. A clinically meaningful improvement is a reduction of ≥1 severity level below baseline maintained across 2 consecutive documented post-surgical quarters; because documentation is sparse and irregular, consecutive refers to consecutive documented quarters and such quarters may be separated by longer intervals. Patients are classified by combining this improvement criterion with the terminal documented trajectory: worsened if the final documented post-surgical quarter is ≥1 severity level above baseline, regardless of any earlier improvement; Responder if a clinically meaningful improvement is present and the final documented quarter is not ≥1 level above baseline; and non-responder otherwise. Time to improvement reflects the number of quarters from VNS surgery to the first quarter of a clinically meaningful improvement; N/A indicates none. Because the surgery quarter counts as post-surgical, improvement detectable in the earliest post-surgical documentation yields a time to improvement of 0 quarters. Ordinal burden scale: −2 = not mentioned (missing, excluded from means); −1 = negated (symptom absent); 0 = present, severity not inferable (not symptom absence); 1 = mild; 2 = moderate; 3 = severe.

#### Cohort-level symptom overview across all 13 symptoms

Table 4 presents median time to improvement across all 13 symptoms. Seizures showed the fastest improvement (median 0.5 quarters, *n* = 8) and gastroesophageal reflux the slowest (18 quarters, *n* = 5). Neuropsychiatric symptoms also improved rapidly, including self-harm (7 quarters, *n* = 11) and suicidal ideation (8 quarters, *n* = 9).

**Table 4.** Median time to clinically meaningful improvement by symptom in VNS test patients.

Symptom	*n* (eligible patients)	*m* (responder patients)	Median time to improvement (quarters)	Range (quarters)	% patients showing improvement (*m*/*n*) (%)
**Seizure**	8	4	0.5	0-2	50.00
**Depression**	14	8	14	5-25	57.14
**Anxiety**	13	6	13	4-23	46.15
**Suicidal ideation**	9	6	8	2-17	66.67
**Pain**	12	6	15	6-26	50.00
**Nausea and vomiting**	8	5	12	4-21	62.50
**Fatigue**	11	6	8	2-18	54.55
**Self-harm**	11	6	7	1-16	54.55
**Headaches**	10	6	11	3-21	60.00
**Gastroesophageal reflux**	5	3	18	8-28	60.00
**Rash**	5	3	14	5-24	60.00
**Paresthesia**	6	2	17	7-27	33.33
**Excessive sleep**	9	4	10	3-19	44.44

Total cohort: *n* = 23; eligible n per symptom reflects the number of patients meeting the minimum documentation threshold of ≥5 clinical notes with symptom mentions in both pre- and post-surgical periods. Clinically meaningful improvement defined as a reduction of at least one severity level below baseline maintained across 2 consecutive documented post-surgical quarters. *m* reflects the number of eligible patients who achieved clinically meaningful improvement. Time is reported in quarters (one quarter = approximately 90 days). Range gives the minimum to maximum time to improvement across responders.

### NSQIP surgical control cohort: symptom trajectory analysis

To test whether VNS-associated improvements reflect neuromodulatory intervention rather than natural disease progression or regression to the mean, longitudinal trajectories were analyzed in the NSQIP surgical control cohort using SME ground-truth annotations. Figure 6 illustrates a representative NSQIP patient whose seizure severity showed no sustained post-surgical decline, unlike the marked improvement in VNS patients (Figure 4); this pattern held across all 13 tracked symptoms, with no systematic post-surgical reduction in NSQIP. Because the cohorts were not matched on diagnosis, baseline severity, follow-up duration, documentation frequency, or surgical indication, this contrast is consistent with, but does not establish, specificity of the VNS-associated improvements. Classification performance was equivalent across VNS and NSQIP test sets (Figure 1; Table S8 in Appendix SA), confirming cross-domain generalizability without corpus-specific overfitting.

**Figure 6.**
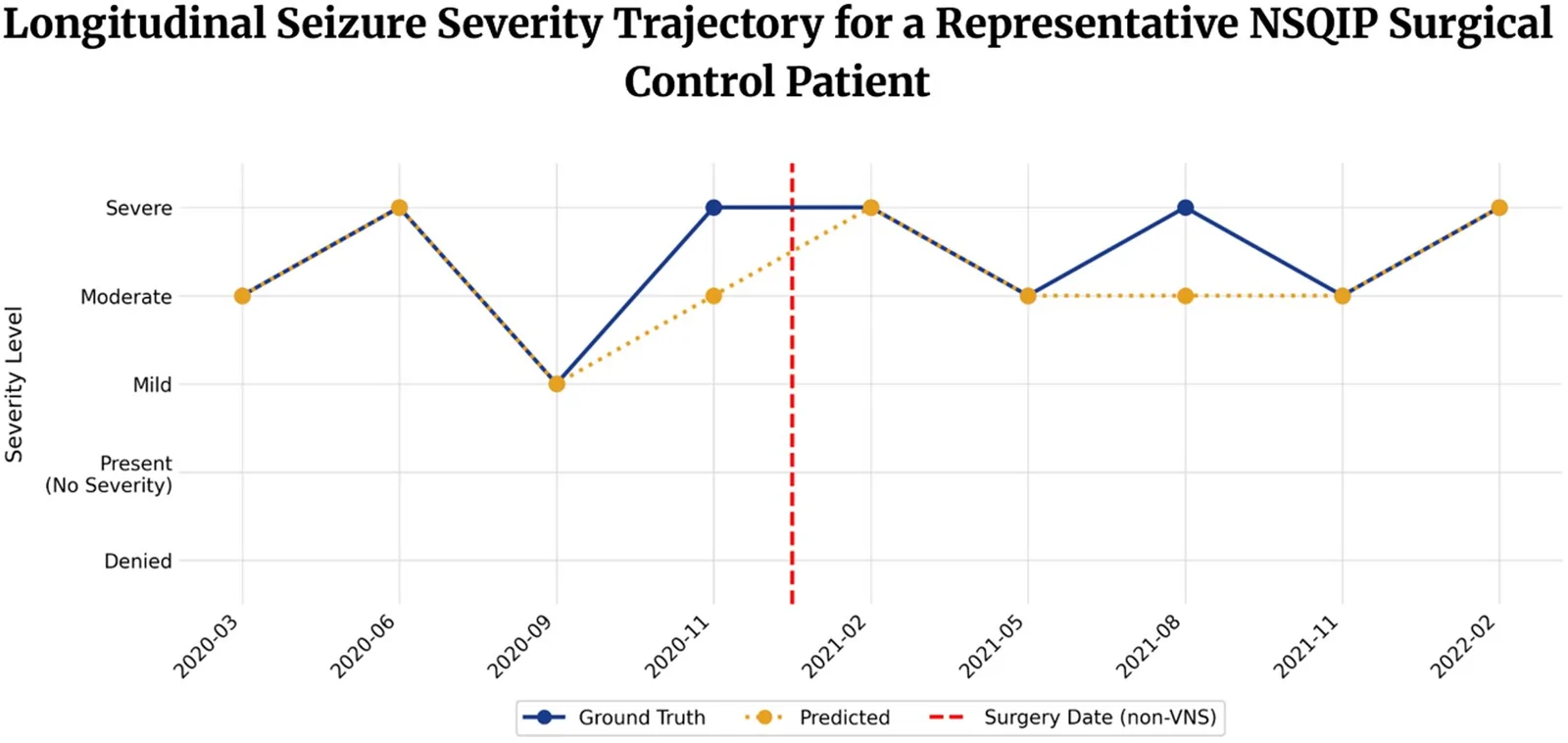
Longitudinal seizure severity trajectory for a representative NSQIP surgical control patient.

### SHAP explainability analysis

#### Inter-symptom severity modeling performance

The OrdinalGBT model outperformed the majority-class baseline MAE for all 5 target symptoms (Table S14 in Appendix SA). Depression showed the largest improvement (baseline MAE: 2.00, model MAE: 0.85) and seizure the smallest (baseline: 1.40, model: 1.06). Suicidal ideation had the highest model MAE (1.35, SD: 0.21), reflecting limited sample availability in 1 severity class.

#### SHAP-based XAI: inter-symptom influence patterns

SHapley Additive exPlanations (SHAP)^14^ summary plots of the OrdinalGBT model recovered clinically coherent inter-symptom dependencies, describing associations among the pipeline’s severity outputs rather than the LLM’s internal decisions. These pattern-level findings, including several counterintuitive associations attributable to documentation artifacts and class imbalance, are detailed in Appendix SA (Figure S3).

## Discussion

### Longitudinal symptom dynamics in VNS

We developed and validated an LLM-based pipeline for extracting, classifying, and longitudinally tracking symptom severity from unstructured clinical notes in VNS patients. To our knowledge, it is the first system to produce structured, patient-level symptom severity trajectories directly from free-text documentation, without patient self-report instruments, curated registries, or structured EHR fields. The pipeline enabled longitudinal monitoring of 13 VNS-relevant symptoms with strong cross-dataset performance, addressing a gap in prior VNS outcome studies, which have focused predominantly on seizure frequency with limited exploration of non-seizure outcomes, quality of life, and longitudinal trajectories.^15^ Seizure severity improved with a median time to clinically meaningful improvement of 0.5 quarters, reflecting improvement already evident in the earliest post-surgical documentation for several patients. Cohort-level analysis revealed substantial heterogeneity: 4 of 8 patients (50%) were classified as responders, 2 showed worsened trajectories, and 2 showed no meaningful change. Patients with sparse documentation could not be meaningfully evaluated, underscoring the dependence of longitudinal AI-based monitoring on documentation completeness.

### Cross-domain generalizability of LLMs

LLMs trained primarily on COVID and PASC notes maintained strong performance on VNS notes, with model size and symptom complexity influencing accuracy more than the source domain, consistent with the per-model results (Table S8 in Appendix SA) and the non-significant corpus effect in the variance decomposition. This aligns with Zhou and Miller and Peng et al,^16^^,^^17^ who demonstrated foundation models transfer across clinical subdomains, supporting unified, cross-condition monitoring on a shared LLM backbone rather than disease-specific models.

This generalizability was not uniform across symptoms, however. Sensitivity for suicidal ideation was lower on NSQIP than on VNS across all models (eg, 0.80 vs 1.00 for LLaMA-3.3-70B; Table S10 in Appendix SA), showing that overall performance and generalization for individual, clinically important symptoms can diverge. This pipeline should not be assumed to generalize to psychiatric risk detection outside its validated population without further evaluation.

### NSQIP surgical control cohort and specificity of VNS effects

The absence of systematic post-surgical symptom severity reduction in NSQIP patients, who underwent major surgery without VNS implantation, is consistent with specificity of the VNS-associated improvements and offers a tentative check against nonspecific surgical effects, increased clinical contact, or regression to the mean. This interpretation is limited, however, by the fact that the NSQIP control was small and was not matched to the VNS cohort on diagnosis, baseline symptom severity, follow-up duration, documentation frequency, or surgical indication. In particular, a control cohort with low baseline symptom burden has limited scope to show further decline, so the absence of a downward trend cannot by itself exclude these alternative explanations. The NSQIP comparison should therefore be read as descriptive supporting context rather than as confirmation of a VNS-specific effect. Equivalent classification performance across both cohorts (Figure 1; Table S8 in Appendix SA) indicates no corpus-specific bias, suggesting potential applicability in non-VNS settings and, with further validation, for post-market surveillance and comparative effectiveness research.

### From clinical narratives to digital biomarkers

Symptom severity has historically been an intermittently collected, patient-reported outcome subject to variability and measurement noise (Topol^18^). We demonstrate that LLMs can instead extract and longitudinally model symptom progression from routine clinical documentation, laying the groundwork for a new class of digital symptom biomarkers. Such approaches may ultimately support real-time treatment monitoring, personalized device programming, and post-market evaluation of neuromodulation therapies.

### Documentation variability and its impact on symptom extraction

Lower performance for subjective symptoms such as pain and anxiety likely reflects a well-documented challenge in clinical NLP: substantial variability in how clinicians document symptoms across patients, providers, and institutions.

Symptom extraction from EHR data is constrained by inconsistencies in terminology, note structure, and granularity;^5^ documentation variability is a primary barrier to generalizable symptom NLP;^19^ physician-level uncertainty drives intra-institutional documentation variation;^20^ and patient-reported symptoms frequently disagree with the record, making clinical notes an imperfect ground truth.^21^ Together, these findings underscore that the performance ceiling of any clinical NLP pipeline is partially determined by documentation consistency, independent of model architecture.

### SHAP-based XAI: clinical interpretation and limitations

The OrdinalGBT model’s outperformance of the baseline MAE indicates that symptom severity in VNS patients is not independent. Most identified patterns aligned with established clinical relationships, lending face validity to the pipeline’s severity outputs.

Counterintuitive findings, such as higher anxiety and self-harm severity predicting lower suicidal ideation severity, likely reflect documentation artifacts: in complex encounters, clinicians may document co-occurring symptoms with differing emphasis, altering how models interpret those features. This highlights a known limitation of SHAP values in clinical settings, which describe feature contribution patterns rather than causal relationships, particularly when documentation biases and correlated features interact.^22^ Class imbalance further contributed to instability for suicidal ideation, where minority severity classes were underrepresented.

Overall, the coherence between LLM-derived severity outputs and structured predictive modeling supports their downstream validity for longitudinal tracking and treatment stratification.

### Limitations and future work

First, data were obtained from a single academic medical center with internally generated annotations, limiting generalizability. Documentation practices vary across institutions and providers,^7^^,^^19–21^ and patient-reported symptoms frequently disagree with the record,^21^ so even SME-annotated notes may incompletely capture true symptom burden (Table 3). Second, severity labels are algorithmically inferred rather than directly measured, requiring external validation. Third, adaptation may be needed for other neuromodulatory interventions (eg, spinal cord or deep brain stimulation), where symptom profiles and documentation patterns differ. Fourth, cohort-level longitudinal analysis was restricted to patients meeting the minimum documentation threshold, limiting representativeness; prospective studies with standardized documentation protocols would increase eligibility. Fifth, our improvement and worsening criteria treat successive documented quarters as consecutive irrespective of the calendar gap, necessary given sparse, irregular documentation but allowing a qualifying pair to span a long interval. Because the surgery quarter counts as post-surgical, improvement in the earliest note yields a time to improvement of zero, contributing to the low seizure median. These timings are therefore documentation-conditioned rather than precise physiological latencies. Sixth, our bootstrap confidence intervals, including for equity subgroups, were computed by note-level resampling and do not account for within-patient clustering. Because each patient contributed 10 to 15 notes, intervals may be narrower than patient-level resampling would yield; because partitions are patient-disjoint and metrics are computed on held-out patients, this affects interval precision rather than point-estimate validity. The concern compounds for the equity analyses, where several subgroups contained very few patients (Table S13 in Appendix SA), so those results should be regarded as exploratory rather than as confirmation of fairness or its absence. This is consistent with prior work on algorithmic bias in clinical AI.^23^^,^^24^ Seventh, our surgical control analysis is limited: the NSQIP cohort was small and was not matched to the VNS cohort on diagnosis, baseline symptom severity, follow-up duration, documentation frequency, or surgical indication, and the trajectory comparison was primarily descriptive (a single representative patient in Figure 6). Because low baseline symptom burden limits scope for further decline, the absence of post-surgical decline in NSQIP provides only weak, non-causal evidence for VNS specificity, and residual confounding cannot be excluded. We did not perform a formal group-by-time analysis, as the unmatched, sparsely documented NSQIP sample would not support a reliable interaction estimate; a matched cohort with a prespecified analysis is needed to establish specificity and is left to future work. Eighth, our summarization fidelity check used 50 summaries reviewed by a single physician and assessed omission qualitatively, without quantifying rates or using multiple reviewers. A larger audit with explicit rates and inter-rater agreement is a direction for future work.

Future work should validate LLM-derived trajectories against patient-reported outcomes and physiological measures, and pursue multi-institutional validation.

## Conclusion

The primary deliverable of this work, fulfilling REVEAL’s Data and Analysis Core Aim 2, is to our knowledge the first structured longitudinal record of symptom severity trajectories in patients undergoing VNS therapy derived directly from free-text clinical documentation without reliance on patient self-report or structured EHR fields. The pipeline operates continuously on new clinical encounters, enabling the dataset to expand with ongoing patient care, supporting downstream predictive modeling, post-market surveillance, and future longitudinal outcome studies. The identification of distinct responder profiles, including sustained improvers, non-responders, and patients with worsened trajectories, provides an early framework for VNS treatment stratification that future predictive models can build upon.

## Supplementary Material

ooag195_Supplementary_Data

## Data Availability

The data underlying this article are available upon reasonable request.
