# Supplementary material for: Longitudinal symptom severity tracking in vagus nerve stimulation patients: a 2-stage LLM-based pipeline with explainable AI
Source: JAMIA Open. 2026 Oct 1;9(5):ooag195. doi: 10.1093/jamiaopen/ooag195 (PMC13630440; doi:10.1093/jamiaopen/ooag195)
Supplement: Supplementary file 1 [file ooag195_supplementary_data.zip › Appendix SA.docx]

**APPENDIX SA**

**Table S1.** Overall symptom prevalence distribution showing count for each symptom followed by its proportion within the respective corpus. Counts reflect all available notes in each source corpus prior to the sampling used to construct the annotated training, validation, and test sets described in the Methods; prevalence in the annotated subsets, particularly the smaller NSQIP test set, may therefore differ, including symptoms with no observed instances in a given partition.

| **Symptom** | **N3C COVID** | **NSQIP** | **MHFV-PASC** | **MHFV-COVID** | **VNS** |
| --- | --- | --- | --- | --- | --- |
| seizure | 7 (0.030) | 3375 (0.019) | 55 (0.017) | 6 (0.029) | 5247 (0.273) |
| depression | 15 (0.063) | 20762 (0.114) | 301 (0.092) | 7 (0.023) | 35554 (0.185) |
| anxiety | 6 (0.025) | 9832 (0.054) | 386 (0.118) | 6 (0.020) | 9976 (0.052) |
| suicidal ideation | 1 (0.004) | 3409 (0.019) | 65 (0.02) | 9 (0.039) | 13861 (0.072) |
| pain | 71 (0.300) | 56696 (0.311) | 670 (0.204) | 670 (0.204) | 21654 (0.113) |
| nausea and vomiting | 63 (0.266) | 22224 (0.122) | 371 (0.113) | 93 (0.309) | 11237 (0.059) |
| fatigue | 25 (0.105) | 9595 (0.053) | 330 (0.101) | 41 (0.136) | 8269 (0.043) |
| self-harm | 0 (0.000) | 711 (0.004) | 41 (0.012) | 1 (0.003) | 4976 (0.026) |
| headaches | 14 (0.059) | 11040 (0.061) | 308 (0.094) | 36 (0.120) | 10628 (0.055) |
| gastroesophageal reflux | 11 (0.046) | 16646 (0.091) | 182 (0.055) | 11 (0.037) | 6242 (0.033) |
| rash | 11 (0.046) | 17299 (0.095) | 403 (0.123) | 54 (0.179) | 8886 (0.046) |
| paresthesia | 9 (0.038) | 8801 (0.048) | 116 (0.035) | 9 (0.030) | 4721 (0.025) |
| excessive sleep | 4 (0.017) | 1957 (0.011) | 54 (0.016) | 10 (0.033) | 3408 (0.018) |

**Table S2.** Symptom prevalence in train, test, and validation datasets.

| **Symptom** | **0 (Absent)** | **1 (Present)** | **2 (Negated)** |
| --- | --- | --- | --- |
| seizure   - Train - Test - Validation | 0.956  0.773  0.689 | 0.034  0.183  0.233 | 0.010  0.043  0.078 |
| depression   - Train - Test - Validation | 0.867  0.641  0.360 | 0.078  0.241  0.436 | 0.054  0.118  0.194 |
| anxiety   - Train - Test - Validation | 0.878  0.712  0.661 | 0.088  0.196  0.252 | 0.034  0.087  0.088 |
| suicidal ideation   - Train - Test - Validation | 0.972  0.790  0.796 | 0.009  0.095  0.107 | 0.019  0.115  0.097 |
| pain   - Train - Test - Validation | 0.956  0.738  0.806 | 0.038  0.198  0.146 | 0.006  0.064  0.048 |
| nausea and vomiting   - Train - Test - Validation | 0.642  0.790  0.718 | 0.100  0.041  0.029 | 0.258  0.169  0.252 |
| fatigue   - Train - Test - Validation | 0.791  0.874  0.893 | 0.163  0.090  0.019 | 0.047  0.035  0.088 |
| self-harm   - Train - Test - Validation | 0.985  0.903  0.922 | 0.004  0.004  0.010 | 0.011  0.093  0.067 |
| headaches   - Train - Test - Validation | 0.810  0.835  0.883 | 0.084  0.097  0.019 | 0.105  0.068  0.097 |
| gastroesophageal reflux   - Train - Test - Validation | 0.982  0.918  0.912 | 0.005  0.033  0.010 | 0.013  0.049  0.078 |
| rash   - Train - Test - Validation | 0.844  0.761  0.796 | 0.026  0.016  0.029 | 0.129  0.223  0.175 |
| paresthesia   - Train - Test - Validation | 0.986  0.932  0.932 | 0.006  0.021  0.010 | 0.008  0.047  0.058 |
| excessive sleep   - Train - Test - Validation | 0.970  0.929  0.901 | 0.013  0.033  0.019 | 0.018  0.037  0.078 |

**Table S3.** Severity prevalence in VNS and NSQIP test datasets.

| **Symptom** | **-2 (Absent)** | **-1 (Negated)** | **0 (Present, No Severity)** | **1 (Mild)** | **2 (Moderate)** | **3 (Severe)** |
| --- | --- | --- | --- | --- | --- | --- |
| Seizure   - VNS - NSQIP | 0.720  0.867 | 0.030  0.133 | 0.080  NaN | 0.030  NaN | 0.050  NaN | 0.060  NaN |
| depression   - VNS - NSQIP | 0.660  0.613 | 0.080  0.207 | 0.070  0.100 | 0.020  0.040 | 0.030  0.027 | 0.110  0.013 |
| anxiety   - VNS - NSQIP | 0.770  0.573 | 0.060  0.173 | 0.090  0.113 | 0.010  0.053 | 0.020  0.040 | 0.020  0.047 |
| suicidal ideation   - VNS - NSQIP | 0.812  0.740 | 0.091  0.233 | 0.019  0.020 | 0.024  0.007 | 0.008  NaN | 0.044  NaN |
| pain   - VNS - NSQIP | 0.688  1.000 | 0.061  NaN | 0.156  NaN | 0.039  NaN | 0.033  NaN | 0.028  NaN |
| nausea and vomiting   - VNS - NSQIP | 0.845  0.560 | 0.127  0.347 | 0.019  0.053 | NaN  0.033 | 0.003  0.007 | 0.006  NaN |
| fatigue   - VNS - NSQIP | 0.856  1.000 | 0.044  NaN | 0.069  NaN | 0.003  NaN | 0.011  NaN | 0.017  NaN |
| self-harm   - VNS - NSQIP | 0.928  0.847 | 0.069  0.147 | NaN  0.007 | NaN  NaN | NaN  NaN | 0.003  NaN |
| headaches   - VNS - NSQIP | 0.881  0.693 | 0.030  0.227 | 0.055  0.060 | 0.008  0.013 | 0.022  0.007 | 0.003  NaN |
| gastroesophageal reflux   - VNS - NSQIP | 0.953  0.767 | 0.030  0.153 | 0.014  0.040 | 0.003  0.020 | NaN  0.020 | NaN  NaN |
| rash   - VNS - NSQIP | 0.812  0.633 | 0.171  0.347 | 0.008  0.013 | 0.003  NaN | 0.003  0.007 | NaN  NaN |
| paresthesia   - VNS - NSQIP | 0.928  1.000 | 0.050  NaN | 0.017  NaN | 0.006  NaN | NaN  NaN | NaN  NaN |
| excessive sleep   - VNS - NSQIP | 0.906  1.000 | 0.050  NaN | 0.025  NaN | 0.003  NaN | 0.014  NaN | 0.003  NaN |

**Table S4.** ANOVA results for symptom classification performance (F1-score).

| **Source** | **Sum of Squares** | **df** | **F** | **PR(>F)** |
| --- | --- | --- | --- | --- |
| C(Corpus) | 0.005 | 4.0 | 0.609 | 0.657 |
| C(validation_group ) | 0.127 | 1.0 | 57.176 | < 0.001 |
| log_tfidf | 0.070 | 1.0 | 31.641 | < 0.001 |
| C(Corpus):log_tfidf | 0.030 | 4 | 3.796 | 0.006 |
| Residual | 0.199 | 99.0 | NaN | NaN |

Analysis of variance (ANOVA) results for the model performance (F1-score) on symptom classification, showing the sum of squares, degrees of freedom, F-statistic, and corresponding p-values.

validation_group denotes the corpus whose held-out notes were used for early stopping during training (levels: VNS, NSQIP), and is distinct from the corpus source of the evaluated notes.

**Table S5.** ANOVA Results for Symptom Severity Assessment Performance (F1-score).

| **Source** | **Sum of Squares** | **df** | **F** | **PR(>F)** |
| --- | --- | --- | --- | --- |
| C(Corpus) | 0.0003 | 4.0 | 0.015 | 0.999 |
| C(validation_group) | 0.046 | 1.0 | 9.093 | 0.003 |
| log_tfidf | 0.002 | 1.0 | 0.418 | 0.519 |
| C(Corpus):log_tfidf | 0.037 | 1 | 7.374 | 0.008 |
| Residual | 0.513 | 102.0 | NaN | NaN |

Analysis of variance (ANOVA) results for the model performance (F1-score) on symptom severity classification, showing the sum of squares, degrees of freedom, F-statistic, and corresponding p-values.

validation_group denotes the corpus whose held-out notes were used for early stopping during training (levels: VNS, NSQIP), and is distinct from the corpus source of the evaluated notes.

**Table S6.** ANOVA Results for Log TF-IDF.

| **Source** | **Sum of Squares** | **df** | **F** | **PR(>F)** |
| --- | --- | --- | --- | --- |
| C(Corpus) | 28.226 | 4.0 | 4.007 | 0.005 |
| C(symptom) | 174.866 | 12.0 | 8.275 | < 0.001 |
| Residual | 163.766 | 93.0 | NaN | NaN |

Analysis of variance (ANOVA) table showing the statistical significance of different predictors for log TF-IDF. The table presents the sum of squares, degrees of freedom, F-statistic, and corresponding p-values.

**Table S7.** η^2^ for Symptom and Severity Classification Performance (F1-score).

| **Source** | **η^2^ (symptom)** | **η^2^ (severity)** |
| --- | --- | --- |
| C (corpus) | 0.013 | 0.0003 |
| C (validation_group) | 0.294 | 0.076 |
| log_tfidf | 0.163 | 0.004 |
| residual | 0.530 | 0.920 |

η^2^ analysis of predictors of model performance (F1-score values) on symptom and severity classification.

validation_group denotes the corpus whose held-out notes were used for early stopping during training (levels: VNS, NSQIP), and is distinct from the corpus source of the evaluated notes.

**Table S8.** Weighted average metrics with 95% CIs for fine-tuned Llama-3.3-70B, Llama-3.2-3B, Llama-3.2-1B models for symptom classification on MHFV-VNS and NSQIP test set.

| **Symptoms** | **MHFV-VNS** | | | **NSQIP** | | |
| --- | --- | --- | --- | --- | --- | --- |
|  | **Precision** | **Recall** | **F1-score** | **Precision** | **Recall** | **F1-score** |
| seizure   - 70B - 3B - 1B | 0.98(0.96-0.99)  0.83(0.79-0.87)  0.78(0.74-0.83) | 0.98(0.96-0.99)  0.83(0.81-0.88)  0.77(0.74-0.82) | 0.98(0.96-0.99)  0.81(0.77-0.86)  0.71(0.66-0.77) | 0.93(0.84-0.97)  0.92(0.78-0.96)  0.84(0.73-0.93) | 0.92(0.89-0.98)  0.92(0.87-0.96)  0.90(0.86-0.96) | 0.91(0.84-0.97)  0.89(0.82-0.95)  0.87(0.79-0.94) |
| depression   - 70B - 3B - 1B | 0.90(0.88-0.93)  0.80(0.77-0.85)  0.76(0.67-0.82) | 0.90(0.87-0.93)  0.79(0.76-0.84)  0.75(0.72-0.81) | 0.89(0.86-0.93)  0.78(0.72-0.82)  0.72(0.67-0.77) | 0.81(0.74-0.87)  0.75(0.65-0.83)  0.72(0.60-0.79) | 0.79(0.71-0.86)  0.73(0.65-0.82)  0.68(0.59-0.76) | 0.77(0.67-0.85)  0.71(0.61-0.81)  0.61(0.50-0.70) |
| anxiety   - 70B - 3B - 1B | 0.96(0.94-0.98)  0.86(0.82-0.90)  0.78(0.73-0.83) | 0.96(0.94-0.98)  0.85(0.82-0.89)  0.83(0.79-0.87) | 0.96(0.93-0.98)  0.84(0.79-0.88)  0.80(0.76-0.85) | 0.86(0.79-0.93)  0.82(0.77-0.88)  0.58(0.46-0.69) | 0.86(0.79-0.92)  0.78(0.70-0.86)  0.69(0.59-0.77) | 0.85(0.77-0.92)  0.76(0.66-0.85)  0.61(0.49-0.71) |
| suicidal ideation   - 70B - 3B - 1B | 0.98(0.97-0.99)  0.89(0.87-0.92)  0.90(0.87-0.93) | 0.98(0.97-0.99)  0.89(0.86-0.92)  0.90(0.87-0.93) | 0.98(0.97-0.99)  0.87(0.83-0.91)  0.89(0.85-0.92) | 0.91(0.89-0.97)  0.88(0.81-0.95)  0.87(0.80-0.94) | 0.90(0.88-0.96)  0.90(0.83-0.95)  0.88(0.82-0.94) | 0.88(0.81-0.96)  0.88(0.80-0.95)  0.87(0.79-0.94) |
| pain   - 70B - 3B - 1B | 0.91(0.88-0.94)  0.78(0.72-0.82)  0.80(0.78-0.82) | 0.90(0.87-0.93)  0.71(0.68-0.76)  0.69(0.66-0.76) | 0.90(0.86-0.93)  0.67(0.58-0.70)  0.66(0.56-0.69) | Not Evaluable* NE  NE | NE  NE  NE | NE  NE  NE |
| nausea and vomiting   - 70B - 3B - 1B | 0.90(0.86-0.93)  0.83(0.78-0.88)  0.85(0.80-0.90) | 0.90(0.88-0.93)  0.85(0.83-0.90)  0.87(0.84-0.91) | 0.89(0.86-0.93)  0.83(0.77-0.87)  0.84(0.79-0.88) | 0.81(0.74-0.87)  0.62(0.52-0.73)  0.64(0.48-0.73) | 0.81(0.72-0.87)  0.64(0.54-0.73)  0.64(0.55-0.72) | 0.79(0.71-0.86)  0.61(0.50-0.72)  0.61(0.49-0.69) |
| fatigue   - 70B - 3B - 1B | 0.92(0.89-0.95)  0.83(0.75-0.86)  0.84(0.80-0.88) | 0.93(0.90-0.95)  0.84(0.82-0.89)  0.85(0.83-0.90) | 0.91(0.88-0.94)  0.83(0.76-0.86)  0.83(0.76-0.86) | NE  NE  NE | NE  NE  NE | NE  NE  NE |
| self-harm   - 70B - 3B - 1B | 0.99(0.98-1.00)  0.96(0.94-0.98)  0.95(0.93-0.98) | 0.99(0.98-1.00)  0.96(0.94-0.98)  0.95(0.93-0.98) | 0.99(0.98-1.00)  0.96(0.94-0.98)  0.95(0.92-0.97) | 0.98(0.95-1.00)  0.97(0.93-1.00)  0.96(0.93-0.99) | 0.98(0.95-1.00)  0.97(0.93-1.00)  0.96(0.92-0.99) | 0.98(0.95-1.00)  0.97(0.93-1.00)  0.96(0.91-0.99) |
| headaches   - 70B - 3B - 1B | 0.96(0.94-0.98)  0.90(0.87-0.92)  0.85(0.78-0.90) | 0.96(0.94-0.98)  0.88(0.86-0.92)  0.87(0.84-0.91) | 0.96(0.94-0.98)  0.88(0.81-0.90)  0.82(0.78-0.87) | 0.87(0.79-0.93)  0.75(0.65-0.85)  0.83(0.71-0.87) | 0.87(0.80-0.92)  0.79(0.72-0.87)  0.78(0.71-0.86) | 0.85(0.77-0.92)  0.74(0.64-0.83)  0.73(0.62-0.82) |
| gastroesophage-al reflux   - 70B - 3B - 1B | 0.98(0.96-0.99)  0.94(0.88-0.96)  0.91(0.86-0.95) | 0.98(0.96-0.99)  0.95(0.93-0.97)  0.95(0.93-0.97) | 0.97(0.95-0.99)  0.94(0.90-0.96)  0.93(0.89-0.96) | 0.95(0.91-0.98)  0.85(0.64-0.90)  0.63(0.51-0.75) | 0.94(0.90-0.98)  0.82(0.74-0.90)  0.78(0.71-0.87) | 0.94(0.88-0.98)  0.76(0.65-0.86)  0.70(0.60-0.80) |
| rash   - 70B - 3B - 1B | 0.93(0.90-0.95)  0.83(0.79-0.87)  0.84(0.79-0.88) | 0.92(0.89-0.95)  0.83(0.80-0.87)  0.83(0.81-0.88) | 0.92(0.89-0.95)  0.83(0.76-0.86)  0.81(0.76-0.86) | 0.91(0.86-0.96)  0.86(0.80-0.92)  0.80(0.72-0.89) | 0.90(0.85-0.95)  0.85(0.78-0.91)  0.82(0.75-0.90) | 0.90(0.84-0.95)  0.86(0.76-0.91)  0.80(0.72-0.88) |
| paresthesia   - 70B - 3B - 1B | 0.99(0.98-1.00)  0.95(0.93-0.97)  0.94(0.83-0.95) | 0.99(0.98-1.00)  0.95(0.92-0.97)  0.90(0.89-0.95) | 0.99(0.98-1.00)  0.95(0.90-0.96)  0.90(0.85-0.93) | NE  NE  NE | NE  NE  NE | NE  NE  NE |
| excessive sleep   - 70B - 3B - 1B | 0.99(0.98-1.00)  0.96(0.81-0.94)  0.87(0.80-0.93) | 0.99(0.98-1.00)  0.91(0.89-0.94)  0.87(0.85-0.92) | 0.99(0.98-1.00)  0.93(0.84-0.92)  0.87(0.83-0.91) | NE  NE  NE | NE  NE  NE | NE  NE  NE |

* Could not be evaluated because there were no mentions of those symptoms in the NSQIP dataset.

**Table S9.** Macro-averaged metrics with 95% CIs for fine-tuned Llama-3.3-70B, Llama-3.2-3B, Llama-3.2-1B models for symptom classification on MHFV-VNS and NSQIP test set.

| **Symptoms** | **MHFV-VNS** | | | **NSQIP** | | |
| --- | --- | --- | --- | --- | --- | --- |
|  | **Precision** | **Recall** | **F1-score** | **Precision** | **Recall** | **F1-score** |
| seizure   - 70B - 3B - 1B | 0.94(0.88-0.98)  0.82(0.75-0.88)  0.72(0.62-0.80) | 0.91(0.84-0.97)  0.77(0.72-0.82)  0.68(0.63-0.72) | 0.92(0.86-0.97)  0.78(0.72-0.83)  0.61(0.53-0.66) | 0.90(0.79-0.97)  0.91(0.53-0.96)  0.60(0.54-0.64) | 0.85(0.77-0.93)  0.68(0.60-0.80)  0.64(0.59-0.66) | 0.85(0.72-0.93)  0.69(0.57-0.83)  0.62(0.57-0.65) |
| depression   - 70B - 3B - 1B | 0.88(0.83-0.92)  0.78(0.71-0.83)  0.73(0.66-0.79) | 0.80(0.75-0.85)  0.70(0.65-0.75)  0.68(0.63-0.73) | 0.82(0.77-0.87)  0.71(0.65-0.77)  0.67(0.61-0.73) | 0.81(0.73-0.90)  0.74(0.63-0.84)  0.69(0.50-0.81) | 0.77(0.68-0.85)  0.72(0.62-0.80)  0.66(0.59-0.72) | 0.76(0.67-0.85)  0.70(0.59-0.79)  0.58(0.48-0.67) |
| anxiety   - 70B - 3B - 1B | 0.93(0.89-0.97)  0.82(0.75-0.88)  0.67(0.56-0.79) | 0.89(0.85-0.94)  0.70(0.65-0.76)  0.64(0.60-0.67) | 0.91(0.87-0.95)  0.72(0.65-0.79)  0.62(0.57-0.67) | 0.85(0.75-0.92)  0.81(0.71-0.89)  0.54(0.41-0.71) | 0.84(0.74-0.93)  0.73(0.63-0.81)  0.62(0.55-0.67) | 0.84(0.75-0.92)  0.71(0.59-0.81)  0.55(0.47-0.63) |
| suicidal ideation   - 70B - 3B - 1B | 0.97(0.94-0.99)  0.81(0.73-0.88)  0.79(0.71-0.86) | 0.96(0.93-0.99)  0.76(0.72-0.80)  0.74(0.69-0.79) | 0.97(0.94-0.99)  0.74(0.68-0.80)  0.72(0.65-0.79) | 0.90(0.53-0.97)  0.66(0.45-0.88)  0.64(0.46-0.82) | 0.66(0.54-0.78)  0.63(0.50-0.76)  0.60(0.46-0.75) | 0.69(0.53-0.83)  0.62(0.47-0.77)  0.61(0.45-0.75) |
| pain   - 70B - 3B - 1B | 0.83(0.75-0.90)  0.63(0.47-0.73)  0.60(0.35-0.71) | 0.79(0.72-0.86)  0.60(0.55-0.64)  0.57(0.51-0.62) | 0.80(0.71-0.86)  0.46(0.40-0.51)  0.41(0.36-0.46) | Not Evaluable* NE  NE | NE  NE  NE | NE  NE  NE |
| nausea and vomiting   - 70B - 3B - 1B | 0.89(0.83-0.92)  0.80(0.72-0.87)  0.75(0.56-0.85) | 0.85(0.79-0.90)  0.76(0.70-0.81)  0.56(0.49-0.64) | 0.86(0.81-0.91)  0.76(0.70-0.82)  0.61(0.51-0.70) | 0.80(0.70-0.89)  0.61(0.50-0.73)  0.60(0.47-0.71) | 0.80(0.71-0.87)  0.63(0.53-0.73)  0.60(0.49-0.71) | 0.78(0.68-0.86)  0.61(0.50-0.71)  0.57(0.45-0.67) |
| fatigue   - 70B - 3B - 1B | 0.91(0.84-0.97)  0.62(0.54-0.69)  0.55(0.40-0.68) | 0.75(0.67-0.83)  0.64(0.58-0.69)  0.61(0.53-0.68) | 0.80(0.73-0.87)  0.63(0.57-0.68)  0.50(0.43-0.60) | NE  NE  NE | NE  NE  NE | NE  NE  NE |
| self-harm   - 70B - 3B - 1B | 0.88(0.66-0.99)  0.76(0.57-0.95)  0.75(0.55-0.94) | 0.99(0.62-0.99)  0.80(0.61-0.98)  0.78(0.59-0.97) | 0.92(0.66-0.99)  0.78(0.59-0.95)  0.76(0.57-0.93) | 0.93(0.77-1.00)  0.91(0.66-1.00)  0.91(0.59-0.99) | 0.91(0.77-1.00)  0.87(0.61-1.00)  0.83(0.56-0.99) | 0.91(0.73-1.00)  0.88(0.63-1.00)  0.86(0.57-0.97) |
| headaches   - 70B - 3B - 1B | 0.91(0.85-0.96)  0.79(0.74-0.85)  0.73(0.56-0.80) | 0.89(0.83-0.94)  0.82(0.76-0.88)  0.66(0.63-0.69) | 0.89(0.84-0.94)  0.80(0.75-0.85)  0.60(0.54-0.66) | 0.87(0.80-0.93)  0.73(0.58-0.88)  0.83(0.76-0.89) | 0.85(0.78-0.91)  0.69(0.62-0.76)  0.76(0.71-0.82) | 0.85(0.76-0.91)  0.67(0.58-0.76)  0.72(0.63-0.81) |
| gastroesophage-al reflux   - 70B - 3B - 1B | 0.90(0.84-0.96)  0.80(0.69-0.91)  0.73(0.59-0.80) | 0.94(0.86-0.99)  0.78(0.68-0.87)  0.65(0.57-0.71) | 0.92(0.85-0.96)  0.79(0.69-0.87)  0.67(0.60-0.74) | 0.91(0.83-0.97)  0.84(0.50-0.91)  0.51(0.42-0.58) | 0.92(0.83-0.99)  0.68(0.62-0.75)  0.61(0.54-0.66) | 0.91(0.83-0.97)  0.65(0.54-0.75)  0.55(0.48-0.60) |
| rash   - 70B - 3B - 1B | 0.93(0.90-0.95)  0.79(0.63-0.87)  0.74(0.62-0.86) | 0.89(0.77-0.96)  0.81(0.66-0.89)  0.75(0.61-0.83) | 0.91(0.91-0.95)  0.80(0.65-0.87)  0.72(0.58-0.81) | 0.85(0.62-0.98)  0.70(0.48-0.90)  0.67(0.43-0.90) | 0.84(0.49-0.91)  0.72(0.50-0.92)  0.62(0.40-0.84) | 0.81(0.51-0.94)  0.71(0.49-0.90)  0.64(0.41-0.83) |
| paresthesia   - 70B - 3B - 1B | 0.88(0.76-0.98)  0.78(0.66-0.87)  0.74(0.38-0.79) | 0.93(0.83-0.99)  0.84(0.73-0.94)  0.64(0.55-0.73) | 0.91(0.80-0.98)  0.81(0.70-0.88)  0.53(0.41-0.63) | NE  NE  NE | NE  NE  NE | NE  NE  NE |
| excessive sleep   - 70B - 3B - 1B | 0.93(0.85-0.99)  0.73(0.65-0.79)  0.33(0.30-0.37) | 0.97(0.91-0.99)  0.89(0.79-0.97)  0.36(0.30-0.44) | 0.95(0.89-0.99)  0.76(0.68-0.83)  0.34(0.30-0.40) | NE  NE  NE | NE  NE  NE | NE  NE  NE |

* Could not be evaluated because there were no mentions of those symptoms in the NSQIP dataset.

**Table S10.** Clinical safety metrics: sensitivity and false-negative rate for suicidal ideation and self-harm.

| **Symptom** | **Cohort** | **Model** | **True Present (n)** | **Detected (TP)** | **Missed (FN)** | **Sensitivity** | **FNR** |
| --- | --- | --- | --- | --- | --- | --- | --- |
| Suicidal ideation | VNS | LLaMA-3.3-70B | 43 | 43 | 0 | 1.00 | 0.00 |
|  |  | LLaMA-3.2-3B | 43 | 42 | 1 | 0.98 | 0.02 |
|  |  | LLaMA-3.2-1B | 43 | 13 | 30 | 0.30 | 0.70 |
|  | NSQIP | LLaMA-3.3-70B | 10 | 8 | 2 | 0.80 | 0.20 |
|  |  | LLaMA-3.2-3B | 10 | 7 | 3 | 0.70 | 0.30 |
|  |  | LLaMA-3.2-1B | 10 | 5 | 5 | 0.50 | 0.50 |
| Self-harm | VNS | LLaMA-3.3-70B | 2 | 2 | 0 | 1.00 | 0.00 |
|  |  | LLaMA-3.2-3B | 2 | 1 | 1 | 0.50 | 0.50 |
|  |  | LLaMA-3.2-1B | 2 | 1 | 1 | 0.50 | 0.50 |
|  | NSQIP | LLaMA-3.3-70B | 4 | 4 | 0 | 1.00 | 0.00 |
|  |  | LLaMA-3.2-3B | 4 | 3 | 1 | 0.75 | 0.25 |
|  |  | LLaMA-3.2-1B | 4 | 3 | 1 | 0.75 | 0.25 |

Sensitivity = recall for the present class. False-negative rate (FNR) = 1 - sensitivity. Self-harm estimates are based on very few present cases (2-4 per cohort) and should be interpreted accordingly.

**Table S11.** Weighted average metrics with 95% CIs for Llama-3.3-70B model on symptom severity assessment in the MHFV-VNS test and NSQIP test set.

| **Symptoms** | **MHFV-VNS** | | | **NSQIP** | | |
| --- | --- | --- | --- | --- | --- | --- |
|  | **Precision** | **Recall** | **F1-score** | **Precision** | **Recall** | **F1-score** |
| seizure | 0.86(0.83-0.91) | 0.86(0.82-0.90) | 0.85(0.81-0.89) | 0.94(0.84-0.97) | 0.93(0.89-0.97) | 0.91(0.84-0.97) |
| depression | 0.79(0.74-0.84) | 0.78(0.73-0.82) | 0.76(0.71-0.80) | 0.82(0.75-0.89) | 0.80(0.72-0.88) | 0.77(0.67-0.86) |
| anxiety | 0.88(0.83-0.91) | 0.85(0.81-0.88) | 0.84(0.80-0.88) | 0.82(0.71-0.88) | 0.76(0.68-0.84) | 0.75(0.66-0.84) |
| suicidal ideation | 0.78(0.72-0.83) | 0.85(0.81-0.88) | 0.81(0.76-0.85) | 0.86(0.80-0.91) | 0.84(0.77-0.90) | 0.79(0.69-0.89) |
| pain | 0.86(0.82-0.90) | 0.81(0.77-0.84) | 0.80(0.76-0.84) | Not Evaluable* | NE | NE |
| nausea and vomiting | 0.90(0.87-0.92) | 0.88(0.84-0.91) | 0.84(0.79-0.88) | 0.77(0.67-0.85) | 0.74(0.65-0.82) | 0.73(0.62-0.81) |
| fatigue | 0.92(0.84-0.95) | 0.90(0.87-0.93) | 0.89(0.85-0.92) | NE | NE | NE |
| self-harm | 0.86(0.81-0.91) | 0.93(0.90-0.95) | 0.89(0.85-0.93) | 0.90(0.77-0.95) | 0.90(0.85-0.95) | 0.87(0.78-0.95) |
| headaches | 0.93(0.88-0.96) | 0.92(0.89-0.94) | 0.91(0.88-0.94) | 0.89(0.82-0.94) | 0.86(0.79-0.92) | 0.85(0.77-0.92) |
| gastroesophageal reflux | 0.97(0.92-0.99) | 0.97(0.95-0.98) | 0.96(0.93-0.98) | 0.91(0.86-0.96) | 0.90(0.85-0.95) | 0.89(0.83-0.96) |
| rash | 0.81(0.69-0.87) | 0.82(0.78-0.86) | 0.76(0.70-0.81) | 0.88(0.84-0.92) | 0.85(0.78-0.91) | 0.84(0.75-0.91) |
| paresthesia | 0.95(0.86-0.97) | 0.94(0.91-0.96) | 0.92(0.88-0.95) | NE | NE | NE |
| excessive sleep | 0.93(0.91-0.96) | 0.94(0.91-0.96) | 0.92(0.89-0.95) | NE | NE | NE |

* Could not be evaluated because there were no mentions of those symptoms in the NSQIP dataset.

**Table S12.** Weighted-averaged metrics for evaluation of LLaMA-3.2-1B/3B and LLaMA-3.3-70B equity for race and gender for symptom classification in MHFV-VNS and NSQIP test sets.

| **Model** | **Parameter** | **MHFV-VNS** | | | **NSQIP** | | |
| --- | --- | --- | --- | --- | --- | --- | --- |
|  |  | **Precision** | **Recall** | **F1-Score** | **Precision** | **Recall** | **F1-Score** |
| LLaMA-3.3-70B | Male  Female  Asian**  Black  White  Others*** | 0.95(0.92,0.97)  0.95(0.91,0.97)  0.99(0.98,1.00)  0.96(0.89,0.99)  0.95(0.92,0.97)  Not Evaluable* | 0.95(0.92,0.97)  0.95(0.92,0.98)  0.99(0.98,1.00)  0.96(0.90,0.98)  0.95(0.92,0.97)  NE | 0.94(0.91,0.97)  0.95(0.91,0.97)  0.99(0.98,1.00)  0.95(0.89,0.99)  0.94(0.91,0.97)  NE | 0.92(0.83,0.99)  0.92(0.85,0.98)  1.00(1.00-1.00)  0.84(0.60,0.97)  0.93(0.87,0.98)  0.94(0.87,1.00) | 0.92(0.84,0.98)  0.92(0.85,0.97)  1.00(1.00-1.00)  0.84(0.66,0.98)  0.92(0.86,0.97)  0.95(0.86,1.00) | 0.91(0.80,0.98)  0.91(0.82,0.97)  1.00(1.00-1.00)  0.81(0.60,0.97)  0.92(0.84,0.97)  0.94(0.84,1.00) |
| LLaMA-3.2-3B | Male  Female  Asian  Black  White  Others | 0.88(0.83,0.94)  0.85(0.79,0.91)  0.89(0.76,1.00)  0.88(0.76,0.99)  0.86(0.80,0.90)  NE | 0.88(0.82,0.94)  0.85(0.79,0.91)  0.85(0.74,0.96)  0.91(0.82,0.99)  0.87(0.83,0.90)  NE | 0.87(0.81,0.93)  0.83(0.77,0.89)  0.83(0.72,0.94)  0.89(0.77,0.98)  0.83(0.79,0.88)  NE | 0.89(0.78,0.97)  0.86(0.75,0.94)  1.00(1.00-1.00)  0.78(0.60,0.94)  0.87(0.76,0.94)  0.89(0.71,1.00) | 0.88(0.78,0.96)  0.86(0.77,0.93)  1.00(1.00-1.00)  0.81(0.63,0.96)  0.87(0.79,0.93)  0.91(0.76,0.98) | 0.86(0.72,0.96)  0.84(0.71,0.92)  1.00(1.00-1.00)  0.77(0.58,0.95)  0.85(0.73,0.92)  0.89(0.72,0.99) |
| LLaMA-3.2-1B | Male  Female  Asian  Black  White  Others | 0.88(0.82,0.92)  0.86(0.80,0.91)  0.88(0.75,0.96)  0.89(0.75,0.98)  0.86(0.77,0.91)  NE | 0.89(0.85,0.93)  0.85(0.79,0.90)  0.84(0.70,0.94) 0.91(0.80,0.98)  0.87(0.81,0.91)  NE | 0.86(0.81,0.91)  0.82(0.75,0.89)  0.82(0.67,0.93)  0.89(0.75,0.98)  0.84(0.76,0.89)  NE | 0.78(0.64,0.91)  0.82(0.69,0.92)  1.00(1.00-1.00)  0.70(0.56,0.76)  0.82(0.70,0.91)  0.89(0.71,1.00) | 0.84(0.72,0.92)  0.85(0.75,0.93)  1.00(1.00-1.00)  0.78(0.60,0.72)  0.84(0.74,0.92)  0.91(0.77,0.98) | 0.79(0.64,0.91)  0.81(0.68,0.91)  1.00(1.00-1.00)  0.72(0.57,0.77)  0.80(0.67,0.89)  0.88(0.72,0.99) |

*Not Evaluable (NE) because VNS test data contained only White, Black and Asian races. **Only 1 patient in the NSQIP test dataset for Male, Asian, and Others subgroups; these single-patient cells reflect within-patient (note-to-note) variability rather than between-patient variability and should not be interpreted as representative of demographic-group performance. *** Others include Latinx and those who denied races.

**Table S13.** Patient and note counts per demographic subgroup in the equity analysis (MHFV-VNS and NSQIP test sets).

| **Subgroup** | **MHFV-VNS test (patients/notes)** | **NSQIP test (patients/notes)** |
| --- | --- | --- |
| Male | 13/ 191 | 1 / 16 |
| Female | 10 / 171 | 5 / 89 |
| Asian | 2 / 30 | 1 / 17 |
| Black | 2 / 30 | 2 / 35 |
| Others* | N/A | 1 / 16 |
| White | 19 / 302 | 2 / 37 |

* Others include Latinx and denied races. N/A: Not Available

**Table S14.** Macro-Averaged MAE for OrdinalGBT Model vs. Baseline (30 trials).

| **Target Symptom** | **MAE** | **SD** | **Baseline MAE** |
| --- | --- | --- | --- |
| seizure | 1.05 | 0.09 | 1.40 |
| depression | 0.85 | 0.06 | 2.00 |
| anxiety | 0.79 | 0.10 | 1.40 |
| suicidal ideation | 1.34 | 0.21 | 2.00 |
| pain | 0.88 | 0.10 | 1.40 |

**Figure S1.** ***(Top)*** The line graph illustrates the longitudinal trajectory of seizure severity for Patient 1 across clinical visits. The solid blue line represents severity labels annotated by subject matter experts (SMEs), while the dotted orange line represents LLM-generated predictions. As shown by the blue line, the patient initially presented between 2011 and 2012 without seizure complaints, but by early 2012 reported severe seizures. In early 2020, the patient again presented with severe seizures and subsequently underwent VNS implantation. Following treatment, seizure severity decreased, as reflected by the blue markers in late 2014, early 2017, and early 2019. The mean pre-surgical seizure severity was **1.80 (~moderate severity)**, which decreased after VNS treatment to **1.00 (mild severity)**, representing a **44.4% reduction in severity**. Similar longitudinal trends are shown for Patient 2 (***middle plot***) and Patient 23 (***bottom plot***).

**Figure S2.** Pre- vs. post-surgical mean **seizure** severity bar chart for 8 VNS patients. For each patient, the mean severity score across all pre-surgical quarters (excluding not-mentioned values) is compared against the mean severity score across all post-surgical quarters. Error bars represent standard deviation. Patients are ordered by magnitude of post-surgical change. Responders show a reduction in mean severity; non-responders and worsened patients are also identified.

**SHAP-based XAI: Inter-symptom influence patterns**

**Seizure** severity was most strongly predicted by **headache** and **excessive** **sleep**, consistent with **epilepsy-sleep**^1^ and **epilepsy-migraine**^2^ comorbidities. A bidirectional **depression-anxiety**^3^ relationship showed asymmetry: negated **anxiety** increased predicted **depression**, while negated **depression** reduced predicted **anxiety**, with **suicidal** **ideation** the next strongest predictor for both.^4^ **Depression** was the dominant predictor of **suicidal** **ideation**,^4^ though higher **anxiety** and **self-harm** severity counterintuitively predicted lower **suicidal** **ideation**.^5^ **Pain** was most strongly predicted by **rash** and **depression**, consistent with chronic **pain-depression** relationships,^6^ with a counterintuitive inverse **anxiety-pain** association.^7^

**Seizure	Depression**

**Anxiety	Suicidal Ideation**

**Pain**

**Figure S3.** The x-axis indicates whether a given symptom increased or decreased the severity of the target symptom. The left y-axis lists the top predictive symptoms ranked by their relative influence on the target symptom, with the most influential symptoms shown at the top and the least influential at the bottom. Each data point represents a single patient visit. Points located to the right of the center line indicate that the corresponding symptom increased the predicted severity of the target symptom, whereas points to the left indicate a decrease in predicted severity. The right y-axis represents symptom severity levels, ranging from 3 (highest severity; red) to -1 (negated/absent; light blue).

**REFERENCES**

[1.](https://www.zotero.org/google-docs/?izkzN5) [Lanigar S, Bandyopadhyay S. Sleep and Epilepsy: A Complex Interplay. *Mo Med*. 2017;114(6):453-457.](https://www.zotero.org/google-docs/?izkzN5)

[2.](https://www.zotero.org/google-docs/?izkzN5) [Kim DW, Lee SK. Headache and Epilepsy. *J Epilepsy Res*. 2017;7(1):7-15. doi:10.14581/jer.17002](https://www.zotero.org/google-docs/?izkzN5)

3. [Kalin NH. The Critical Relationship Between Anxiety and Depression. *AJP*. 2020;177(5):365-367. doi:10.1176/appi.ajp.2020.20030305](https://www.zotero.org/google-docs/?izkzN5)

[4.](https://www.zotero.org/google-docs/?izkzN5) [Li J, Zhang Y, Siu Man Chan B, et al. Associations between anxiety, depression, and risk of suicidal behaviors in Chinese medical college students. *Front Psychiatry*. 2022;13:1012298. doi:10.3389/fpsyt.2022.1012298](https://www.zotero.org/google-docs/?izkzN5)

[5.](https://www.zotero.org/google-docs/?izkzN5) [Brankovic A, Rolls D, Boyle J, Niven P, Khanna S. Identifying patients at risk of unplanned re-hospitalisation using statewide electronic health records. *Sci Rep*. 2022;12(1):16592. doi:10.1038/s41598-022-20907-z](https://www.zotero.org/google-docs/?izkzN5)

[6.](https://www.zotero.org/google-docs/?izkzN5) [Sheng J, Liu S, Wang Y, Cui R, Zhang X. The Link between Depression and Chronic Pain: Neural Mechanisms in the Brain. *Neural Plast*. 2017;2017:9724371. doi:10.1155/2017/9724371](https://www.zotero.org/google-docs/?izkzN5)

[7.](https://www.zotero.org/google-docs/?izkzN5) [Woo AK. Depression and Anxiety in Pain. *Rev Pain*. 2010;4(1):8-12. doi:10.1177/204946371000400103](https://www.zotero.org/google-docs/?izkzN5)
