# Supplementary material for: Longitudinal symptom severity tracking in vagus nerve stimulation patients: a 2-stage LLM-based pipeline with explainable AI
Source: JAMIA Open. 2026 Oct 1;9(5):ooag195. doi: 10.1093/jamiaopen/ooag195 (PMC13630440; doi:10.1093/jamiaopen/ooag195)
Supplement: Supplementary file 1 [file ooag195_supplementary_data.zip › Appendix SB.docx]

**APPENDIX SB**

**LLM FINE-TUNING FOR SYMPTOM CLASSIFICATION**

Fine-tuning updated only the final classification layer, using cross-entropy loss; full training configuration is given in Table S15. At inference, output logits were converted to class probabilities with a softmax function.^1^

**Table S15.** Stage 1 training configuration (symptom classification).

| **Setting** | **Value** |
| --- | --- |
| Fine-tuning Method | Final classification layer only; backbone frozen; LoRA not used |
| Base Models | LLaMA-3.2-1B, LLaMA-3.2-3B, LLaMA-3.3-70B |
| Output Layer | 39 neurons (13 symptoms × 3 status classes) |
| Trainable Parameters | 1B: 79,911; 3B: 119,847; 70B: 319,527 |
| Loss | Cross-entropy^2^ |
| Optimizer | AdamW^3^ |
| Learning Rate | 2e-5 |
| Weight Decay | 0.01 |
| Dropout | 0.1 |
| Batch Size | 8 |
| Max Epochs | 50 |
| Model Selection | Early stopping on validation set; monitored cross-entropy, weighted precision, recall, F1, AUROC; checkpoint saved on any validation-metric improvement |
| Context Length | 4096 |
| Truncation Strategy | Right truncation, though summaries always fell within this limit |
| Random Seeds | 42 |
| Hardware | 4 x A100 GPUs, secure supercomputing environment, University of Minnesota |
| Quantization | 4-bit backbone for 70B^4^ |

**LLM PROMPTING FOR SEVERITY ASSESSMENT**

A pre-trained LLaMA-3.3-70B model with generated knowledge prompting was used for severity assessment to assess severity levels of identified symptoms. A standardized severity scoring system with three distinct levels was used: mild (score: 1), moderate (score: 2), and severe (score: 3). In this approach, the model was first provided with contextual information about the identified symptoms and then prompted to assess their severity. A typical prompt followed this structure: "*Given the following symptoms identified in this summarized clinical note: [list of symptoms], please assess the severity of each symptom as either mild, moderate, or severe and provide your assessment in JSON format."*

The model’s output was then normalized and validated. This post-processing step involved several stages to convert the textual output into a structured and clinically useful format. First, the raw text output from the model, which included a JSON-like structure embedded within a natural language response (e.g., "*here are the symptom severities: {seizure: mild, headaches: moderate, nausea: severe}*"), was parsed using regular expressions to extract key-value pairs representing symptoms and their corresponding severities. Once the mapping was extracted, the severity descriptions (e.g., "mild," "moderate," "severe") were then mapped to numerical scores (1, 2, or 3, respectively) to facilitate easier analysis and integration into downstream applications. To ensure robustness of the output parsing pipeline, prompt engineering was used to constrain the model's responses to predefined severity labels (mild, moderate, severe), effectively eliminating unrecognized label outputs. Similarly, since the prompt explicitly provided the list of symptoms and requested severity for each, missing symptom outputs were not observed. However, a small number of cases produced malformed JSON output that could not be resolved through prompt engineering alone; these cases were flagged and reviewed manually to ensure annotation completeness.

**SUMMARY NOTES VERSUS FULL NOTES**

To evaluate the effectiveness of using clinical note summarization, we compared symptom classification performance between full-text and summarized notes using the fine-tuned LLaMA-3.2-3B model. This comparison was designed to determine whether summarized notes could achieve comparable or improved performance in symptom classification and severity assessment while increasing processing efficiency.

Overall, summarized notes achieved performance comparable to or better than full-text notes (Table S16 and Figure S4). Symptoms such as **anxiety** and **nausea and vomiting** showed improved performance with summarized notes. For example, the F1-score for **anxiety** increased from 0.75 using full notes to 0.85 with summarized notes. This improvement may reflect the ability of summarization to remove extraneous information and emphasize clinically relevant symptom descriptions.

**Summarization fidelity audit.** To verify that summarization did not drop clinically relevant content, 50 summaries (10 randomly selected from each of the five corpora) were audited by an attending physician (MD, MS) who compared each summary side-by-side against its full source note. No clinically relevant omissions were identified in any of the 50 audited summaries. The audit assessed omission of clinically relevant content; hallucination of unsupported content was not separately quantified.

**Table S16.** Weighted average metrics for fine-tuned Llama-3.2-3B model for symptom classification on VNS test set summary notes vs full notes.

| **Symptoms** | **Precision** | **Recall** | **F1-score** |
| --- | --- | --- | --- |
| seizure   - summary notes - full notes | 0.83  0.81 | 0.84  0.85 | 0.81  0.83 |
| depression   - summary notes - full notes | 0.81  0.78 | 0.80  0.77 | 0.77  0.71 |
| anxiety   - summary notes - full notes | 0.87  0.72 | 0.86  0.80 | 0.85  0.75 |
| suicidal ideation   - summary notes - full notes | 0.89  0.85 | 0.89  0.90 | 0.87  0.87 |
| pain   - summary notes - full notes | 0.80  0.81 | 0.72  0.87 | 0.64  0.82 |
| nausea and vomiting   - summary notes - full notes | 0.83  0.63 | 0.86  0.65 | 0.82  0.64 |
| fatigue   - summary notes - full notes | 0.81  0.70 | 0.86  0.78 | 0.81  0.73 |
| self-harm   - summary notes - full notes | 0.96  0.91 | 0.96  0.94 | 0.96  0.92 |
| headaches   - summary notes - full notes | 0.90  0.73 | 0.89  0.81 | 0.86  0.77 |
| gastroesophageal reflux   - summary notes - full notes | 0.93  0.85 | 0.96  0.90 | 0.94  0.87 |
| rash   - summary notes - full notes | 0.83  0.77 | 0.84  0.78 | 0.81  0.77 |
| paresthesia   - summary notes - full notes | 0.95  0.92 | 0.95  0.95 | 0.94  0.94 |
| excessive sleep   - summary notes - full notes | 0.92  0.88 | 0.92  0.91 | 0.88  0.90 |

**Figure S4.** Performance comparison of the Llama-3.2-3B model on symptom classification using note summaries versus full text notes. F1-scores across symptoms, with green markers indicate cases where summary notes outperform full notes.

In contrast, some symptoms, such as **pain**, showed slightly lower performance with summarized notes, with the F1-score decreasing from 0.82 for full-text notes to 0.64 for summarized notes. This reduction may result from the loss of contextual details during summarization that are important for accurate symptom classification.

**Design tradeoffs: summarization versus clinical accuracy**

We also observed that LLM generated summarization introduces an important tradeoff between efficiency and contextual fidelity.

Summarized clinical notes improved classification performance for some symptoms, such as **anxiety** and **nausea and vomiting**, but reduced performance for others, particularly **pain**, where clinically important contextual details may be lost during summarization. These findings are consistent with Alambo *et al.* (2022) and Van Veen *et al.* (2024), who noted that clinical summarization can compromise factual accuracy and obscure contextual information necessary for accurate clinical inference.^5,6^ As LLMs are increasingly integrated into EHR systems and real-time clinical decision support systems, our results highlight the importance of carefully evaluating summarization and compression strategies, particularly in longitudinal symptom-tracking applications where subtle temporal and contextual signals may carry significant clinical meaning.

**CORPORA PERFORMANCE EFFECTS: TF-IDF COMPUTATION**

TF-IDF quantifies the relative importance of terms across documents by combining within-document term frequency (TF) with corpus-wide inverse document frequency (IDF), enabling identification of symptom-specific keywords distinctive to each corpus. IDF values were calculated across the combined corpora, while TF values were calculated per note within each corpus; mean symptom-specific TF-IDF values were used in all ANOVA models. Additional ANOVA models assessed whether TF-IDF values differed significantly by corpus and symptom.

**INTER-SYMPTOM MODELING (ORDINALGBT) AND EXPLAINABILITY**

The dataset comprised 607 VNS patient interactions across 13 symptoms, encoded on the ordinal burden scale from -1 (negated) through 3 (severe); not-mentioned (-2) entries were treated as missing and excluded, and the negated level (-1) was retained as the lowest-burden ordinal category consistent with the scale's clinical ordering. Five symptoms meeting eligibility criteria (all severity classes represented, at least 5 samples per class) served as targets (seizure, depression, anxiety, suicidal ideation, and pain); the remaining eight served as input features. Hyperparameter optimization used Optuna with 30 trials per symptom and 5-fold cross-validation grouped by patient (GroupKFold), so that all interactions from a given patient fell within a single fold, evaluated by macro-averaged mean absolute error (MAE). Class weights addressed class imbalance. SHAP values were averaged across 30 randomized trials and compared against a majority-class baseline MAE.

**REFERENCES**

1.	Nwankpa C, Ijomah W, Gachagan A, Marshall S. Activation Functions: Comparison of trends in Practice and Research for Deep Learning. *arXiv*. Preprint posted online 2018. doi:10.48550/ARXIV.1811.03378

2.	Mao A, Mohri M, Zhong Y. Cross-Entropy Loss Functions: Theoretical Analysis and Applications. *arXiv*. Preprint posted online 2023. doi:10.48550/ARXIV.2304.07288

3.	Kingma DP, Ba J. Adam: A Method for Stochastic Optimization. *arXiv*. Preprint posted online 2014. doi:10.48550/ARXIV.1412.6980

4.	Dettmers T, Pagnoni A, Holtzman A, Zettlemoyer L. QLoRA: Efficient Finetuning of Quantized LLMs. Published online 2023. doi:10.48550/ARXIV.2305.14314

5.	Alambo A, Banerjee T, Thirunarayan K, Cajita M. Improving the Factual Accuracy of Abstractive Clinical Text Summarization using Multi-Objective Optimization. In: *2022 44th Annual International Conference of the IEEE Engineering in Medicine & Biology Society (EMBC)*. 2022:1615-1618. doi:10.1109/EMBC48229.2022.9871798

6.	Van Veen D, Van Uden C, Blankemeier L, et al. Clinical Text Summarization: Adapting Large Language Models Can Outperform Human Experts. *Research Square*. Preprint posted online October 30, 2023:rs. 3.rs-3483777. doi:10.21203/rs.3.rs-3483777/v1
