# Supplementary material for: Longitudinal symptom severity tracking in vagus nerve stimulation patients: a 2-stage LLM-based pipeline with explainable AI
Source: JAMIA Open. 2026 Oct 1;9(5):ooag195. doi: 10.1093/jamiaopen/ooag195 (PMC13630440; doi:10.1093/jamiaopen/ooag195)
Supplement: Supplementary file 1 [file ooag195_supplementary_data.zip › Appendix SC.docx]

**Table S17.** Per-Class Precision, Recall, and F1-Score by Symptom, Dataset, and Model.

**Seizure**

| **Model (Dataset)** | **Class** | **n** | **Precision** | **Recall** | **F1-score** |
| --- | --- | --- | --- | --- | --- |
| LLaMA-3.3-70B (VNS) | Absent | 259 | 0.996 | 0.992 | 0.994 |
|  | Present | 83 | 0.954 | 1.000 | 0.976 |
|  | Negated | 20 | 0.882 | 0.750 | 0.811 |
| LLaMA-3.2-3B (VNS) | Absent | 259 | 0.840 | 0.954 | 0.893 |
|  | Present | 83 | 0.800 | 0.434 | 0.562 |
|  | Negated | 20 | 0.826 | 0.950 | 0.884 |
| LLaMA-3.2-1B (VNS) | Absent | 259 | 0.792 | 0.954 | 0.865 |
|  | Present | 83 | 0.812 | 0.157 | 0.263 |
|  | Negated | 20 | 0.559 | 0.950 | 0.704 |
| LLaMA-3.3-70B (NSQIP) | Absent | 80 | 0.930 | 1.000 | 0.964 |
|  | Present | 18 | 1.000 | 0.556 | 0.714 |
|  | Negated | 7 | 0.778 | 1.000 | 0.875 |
| LLaMA-3.2-3B (NSQIP) | Absent | 80 | 0.940 | 0.975 | 0.957 |
|  | Present | 18 | 0.810 | 0.944 | 0.872 |
|  | Negated | 7 | 1.000 | 0.143 | 0.250 |
| LLaMA-3.2-1B (NSQIP) | Absent | 80 | 0.907 | 0.975 | 0.940 |
|  | Present | 18 | 0.895 | 0.944 | 0.919 |
|  | Negated | 7 | 0.000 | 0.000 | 0.000 |

**Depression**

| **Model (Dataset)** | **Class** | **n** | **Precision** | **Recall** | **F1-score** |
| --- | --- | --- | --- | --- | --- |
| LLaMA-3.3-70B (VNS) | Absent | 206 | 0.919 | 0.985 | 0.951 |
|  | Present | 105 | 0.875 | 0.933 | 0.903 |
|  | Negated | 51 | 0.862 | 0.490 | 0.625 |
| LLaMA-3.2-3B (VNS) | Absent | 206 | 0.863 | 0.859 | 0.861 |
|  | Present | 105 | 0.682 | 0.857 | 0.759 |
|  | Negated | 51 | 0.800 | 0.392 | 0.526 |
| LLaMA-3.2-1B (VNS) | Absent | 206 | 0.770 | 0.961 | 0.855 |
|  | Present | 105 | 0.778 | 0.333 | 0.467 |
|  | Negated | 51 | 0.650 | 0.765 | 0.703 |
| LLaMA-3.3-70B (NSQIP) | Absent | 57 | 0.768 | 0.930 | 0.841 |
|  | Present | 33 | 0.944 | 0.515 | 0.667 |
|  | Negated | 15 | 0.722 | 0.867 | 0.788 |
| LLaMA-3.2-3B (NSQIP) | Absent | 57 | 0.725 | 0.877 | 0.794 |
|  | Present | 33 | 0.824 | 0.424 | 0.560 |
|  | Negated | 15 | 0.684 | 0.867 | 0.765 |
| LLaMA-3.2-1B (NSQIP) | Absent | 57 | 0.711 | 0.947 | 0.812 |
|  | Present | 33 | 0.800 | 0.121 | 0.211 |
|  | Negated | 15 | 0.583 | 0.933 | 0.718 |

**Anxiety**

| **Model (Dataset)** | **Class** | **n** | **Precision** | **Recall** | **F1-score** |
| --- | --- | --- | --- | --- | --- |
| LLaMA-3.3-70B (VNS) | Absent | 235 | 0.975 | 1.000 | 0.987 |
|  | Present | 88 | 0.955 | 0.955 | 0.955 |
|  | Negated | 39 | 0.879 | 0.744 | 0.806 |
| LLaMA-3.2-3B (VNS) | Absent | 235 | 0.921 | 0.940 | 0.931 |
|  | Present | 88 | 0.701 | 0.852 | 0.769 |
|  | Negated | 39 | 0.867 | 0.333 | 0.481 |
| LLaMA-3.2-1B (VNS) | Absent | 235 | 0.851 | 0.923 | 0.886 |
|  | Present | 88 | 0.827 | 0.920 | 0.871 |
|  | Negated | 39 | 0.333 | 0.077 | 0.125 |
| LLaMA-3.3-70B (NSQIP) | Absent | 67 | 0.906 | 0.866 | 0.885 |
|  | Present | 27 | 0.742 | 0.852 | 0.793 |
|  | Negated | 11 | 0.900 | 0.818 | 0.857 |
| LLaMA-3.2-3B (NSQIP) | Absent | 67 | 0.775 | 0.925 | 0.844 |
|  | Present | 27 | 1.000 | 0.370 | 0.541 |
|  | Negated | 11 | 0.667 | 0.909 | 0.769 |
| LLaMA-3.2-1B (NSQIP) | Absent | 67 | 0.721 | 0.925 | 0.810 |
|  | Present | 27 | 0.200 | 0.037 | 0.062 |
|  | Negated | 11 | 0.714 | 0.909 | 0.800 |

**Suicidal Ideation**

| **Model (Dataset)** | **Class** | **n** | **Precision** | **Recall** | **F1-score** |
| --- | --- | --- | --- | --- | --- |
| LLaMA-3.3-70B (VNS) | Absent | 266 | 0.985 | 0.992 | 0.989 |
|  | Present | 43 | 0.977 | 1.000 | 0.989 |
|  | Negated | 53 | 0.960 | 0.906 | 0.932 |
| LLaMA-3.2-3B (VNS) | Absent | 266 | 0.939 | 0.989 | 0.963 |
|  | Present | 43 | 0.689 | 0.977 | 0.808 |
|  | Negated | 53 | 0.810 | 0.321 | 0.459 |
| LLaMA-3.2-1B (VNS) | Absent | 266 | 0.978 | 0.985 | 0.981 |
|  | Present | 43 | 0.765 | 0.302 | 0.433 |
|  | Negated | 53 | 0.649 | 0.943 | 0.769 |
| LLaMA-3.3-70B (NSQIP) | Absent | 85 | 0.914 | 1.000 | 0.955 |
|  | Present | 10 | 0.800 | 0.800 | 0.800 |
|  | Negated | 10 | 1.000 | 0.200 | 0.333 |
| LLaMA-3.2-3B (NSQIP) | Absent | 85 | 0.966 | 1.000 | 0.983 |
|  | Present | 10 | 0.538 | 0.700 | 0.609 |
|  | Negated | 10 | 0.500 | 0.200 | 0.286 |
| LLaMA-3.2-1B (NSQIP) | Absent | 85 | 0.966 | 1.000 | 0.983 |
|  | Present | 10 | 0.455 | 0.500 | 0.476 |
|  | Negated | 10 | 0.500 | 0.300 | 0.375 |

**Pain**

| **Model (Dataset)** | **Class** | **n** | **Precision** | **Recall** | **F1-score** |
| --- | --- | --- | --- | --- | --- |
| LLaMA-3.3-70B (VNS) | Absent | 267 | 0.965 | 0.921 | 0.943 |
|  | Present | 72 | 0.739 | 0.944 | 0.829 |
|  | Negated | 23 | 0.800 | 0.522 | 0.632 |
| LLaMA-3.2-3B (VNS) | Absent | 267 | 0.819 | 0.880 | 0.848 |
|  | Present | 72 | 0.800 | 0.056 | 0.104 |
|  | Negated | 23 | 0.286 | 0.870 | 0.430 |
| LLaMA-3.2-1B (VNS) | Absent | 267 | 0.790 | 0.933 | 0.856 |
|  | Present | 72 | 1.000 | 0.014 | 0.027 |
|  | Negated | 23 | 0.065 | 0.130 | 0.087 |
| NSQIP | Not Evaluable | - | - | - | - |

**Nausea and Vomiting**

| **Model (Dataset)** | **Class** | **n** | **Precision** | **Recall** | **F1-score** |
| --- | --- | --- | --- | --- | --- |
| LLaMA-3.3-70B (VNS) | Absent | 277 | 0.914 | 0.964 | 0.938 |
|  | Present | 16 | 0.938 | 0.938 | 0.938 |
|  | Negated | 69 | 0.833 | 0.652 | 0.732 |
| LLaMA-3.2-3B (VNS) | Absent | 277 | 0.866 | 0.953 | 0.907 |
|  | Present | 16 | 0.833 | 0.938 | 0.882 |
|  | Negated | 69 | 0.718 | 0.406 | 0.519 |
| LLaMA-3.2-1B (VNS) | Absent | 277 | 0.888 | 1.000 | 0.941 |
|  | Present | 16 | 0.600 | 0.188 | 0.286 |
|  | Negated | 69 | 0.778 | 0.507 | 0.614 |
| LLaMA-3.3-70B (NSQIP) | Absent | 57 | 0.806 | 0.947 | 0.871 |
|  | Present | 13 | 0.750 | 0.923 | 0.828 |
|  | Negated | 35 | 0.864 | 0.543 | 0.667 |
| LLaMA-3.2-3B (NSQIP) | Absent | 57 | 0.662 | 0.789 | 0.720 |
|  | Present | 13 | 0.625 | 0.769 | 0.690 |
|  | Negated | 35 | 0.571 | 0.343 | 0.429 |
| LLaMA-3.2-1B (NSQIP) | Absent | 57 | 0.676 | 0.842 | 0.750 |
|  | Present | 13 | 0.500 | 0.692 | 0.581 |
|  | Negated | 35 | 0.625 | 0.286 | 0.392 |

**Fatigue**

| **Model (Dataset)** | **Class** | **n** | **Precision** | **Recall** | **F1-score** |
| --- | --- | --- | --- | --- | --- |
| LLaMA-3.3-70B (VNS) | Absent | 316 | 0.934 | 0.991 | 0.962 |
|  | Present | 33 | 0.800 | 0.364 | 0.500 |
|  | Negated | 13 | 1.000 | 0.923 | 0.960 |
| LLaMA-3.2-3B (VNS) | Absent | 316 | 0.904 | 0.924 | 0.914 |
|  | Present | 33 | 0.120 | 0.091 | 0.103 |
|  | Negated | 13 | 0.857 | 0.923 | 0.889 |
| LLaMA-3.2-1B (VNS) | Absent | 316 | 0.905 | 0.934 | 0.919 |
|  | Present | 33 | 0.400 | 0.061 | 0.105 |
|  | Negated | 13 | 0.355 | 0.846 | 0.500 |
| NSQIP | Not Evaluable |  |  |  |  |

**Self-harm**

| **Model (Dataset)** | **Class** | **n** | **Precision** | **Recall** | **F1-score** |
| --- | --- | --- | --- | --- | --- |
| LLaMA-3.3-70B (VNS) | Absent | 317 | 1.000 | 1.000 | 1.000 |
|  | Present | 2 | 0.667 | 1.000 | 0.800 |
|  | Negated | 43 | 1.000 | 0.977 | 0.988 |
| LLaMA-3.2-3B (VNS) | Absent | 317 | 0.987 | 0.968 | 0.978 |
|  | Present | 2 | 0.500 | 0.500 | 0.500 |
|  | Negated | 43 | 0.816 | 0.930 | 0.870 |
| LLaMA-3.2-1B (VNS) | Absent | 317 | 0.981 | 0.962 | 0.971 |
|  | Present | 2 | 0.500 | 0.500 | 0.500 |
|  | Negated | 43 | 0.776 | 0.884 | 0.826 |
| LLaMA-3.3-70B (NSQIP) | Absent | 93 | 0.989 | 1.000 | 0.995 |
|  | Present | 4 | 0.800 | 1.000 | 0.889 |
|  | Negated | 8 | 1.000 | 0.750 | 0.857 |
| LLaMA-3.2-3B (NSQIP) | Absent | 93 | 0.979 | 0.989 | 0.984 |
|  | Present | 4 | 0.750 | 0.750 | 0.750 |
|  | Negated | 8 | 1.000 | 0.875 | 0.933 |
| LLaMA-3.2-1B (NSQIP) | Absent | 93 | 0.979 | 0.989 | 0.984 |
|  | Present | 4 | 1.000 | 0.750 | 0.857 |
|  | Negated | 8 | 0.750 | 0.750 | 0.750 |

**Headaches**

| **Model (Dataset)** | **Class** | **n** | **Precision** | **Recall** | **F1-score** |
| --- | --- | --- | --- | --- | --- |
| LLaMA-3.3-70B (VNS) | Absent | 291 | 0.979 | 0.983 | 0.981 |
|  | Present | 41 | 0.911 | 1.000 | 0.953 |
|  | Negated | 30 | 0.840 | 0.700 | 0.764 |
| LLaMA-3.2-3B (VNS) | Absent | 291 | 0.943 | 0.904 | 0.923 |
|  | Present | 41 | 0.519 | 0.683 | 0.589 |
|  | Negated | 30 | 0.931 | 0.900 | 0.915 |
| LLaMA-3.2-1B (VNS) | Absent | 291 | 0.904 | 0.976 | 0.939 |
|  | Present | 41 | 0.667 | 0.049 | 0.091 |
|  | Negated | 30 | 0.644 | 0.967 | 0.773 |
| LLaMA-3.3-70B (NSQIP) | Absent | 50 | 0.860 | 0.980 | 0.916 |
|  | Present | 33 | 0.955 | 0.636 | 0.764 |
|  | Negated | 22 | 0.808 | 0.955 | 0.875 |
| LLaMA-3.2-3B (NSQIP) | Absent | 50 | 0.790 | 0.980 | 0.875 |
|  | Present | 33 | 0.833 | 0.909 | 0.870 |
|  | Negated | 22 | 0.571 | 0.182 | 0.276 |
| LLaMA-3.2-1B (NSQIP) | Absent | 50 | 0.769 | 1.000 | 0.870 |
|  | Present | 33 | 1.000 | 0.303 | 0.465 |
|  | Negated | 22 | 0.733 | 1.000 | 0.846 |

**Gastroesophageal Reflux (GERD)**

| **Model (Dataset)** | **Class** | **n** | **Precision** | **Recall** | **F1-score** |
| --- | --- | --- | --- | --- | --- |
| LLaMA-3.3-70B (VNS) | Absent | 334 | 0.994 | 0.982 | 0.988 |
|  | Present | 11 | 1.000 | 0.909 | 0.952 |
|  | Negated | 17 | 0.727 | 0.941 | 0.821 |
| LLaMA-3.2-3B (VNS) | Absent | 334 | 0.970 | 0.976 | 0.973 |
|  | Present | 11 | 0.909 | 0.909 | 0.909 |
|  | Negated | 17 | 0.533 | 0.471 | 0.500 |
| LLaMA-3.2-1B (VNS) | Absent | 334 | 0.951 | 0.991 | 0.971 |
|  | Present | 11 | 1.000 | 0.909 | 0.952 |
|  | Negated | 17 | 0.250 | 0.059 | 0.095 |
| LLaMA-3.3-70B (NSQIP) | Absent | 71 | 0.958 | 0.958 | 0.958 |
|  | Present | 14 | 1.000 | 0.857 | 0.923 |
|  | Negated | 20 | 0.864 | 0.950 | 0.905 |
| LLaMA-3.2-3B (NSQIP) | Absent | 71 | 0.843 | 0.986 | 0.909 |
|  | Present | 14 | 0.684 | 0.929 | 0.788 |
|  | Negated | 20 | 1.000 | 0.150 | 0.261 |
| LLaMA-3.2-1B (NSQIP) | Absent | 71 | 0.787 | 0.986 | 0.875 |
|  | Present | 14 | 0.750 | 0.857 | 0.800 |
|  | Negated | 20 | 0.000 | 0.000 | 0.000 |

**Rash**

| **Model (Dataset)** | **Class** | **n** | **Precision** | **Recall** | **F1-score** |
| --- | --- | --- | --- | --- | --- |
| LLaMA-3.3-70B (VNS) | Absent | 261 | 0.972 | 0.931 | 0.951 |
|  | Present | 6 | 1.000 | 0.833 | 0.909 |
|  | Negated | 95 | 0.822 | 0.926 | 0.871 |
| LLaMA-3.2-3B (VNS) | Absent | 261 | 0.903 | 0.854 | 0.878 |
|  | Present | 6 | 0.833 | 0.833 | 0.833 |
|  | Negated | 95 | 0.661 | 0.758 | 0.706 |
| LLaMA-3.2-1B (VNS) | Absent | 261 | 0.837 | 0.962 | 0.895 |
|  | Present | 6 | 0.556 | 0.833 | 0.667 |
|  | Negated | 95 | 0.849 | 0.474 | 0.608 |
| LLaMA-3.3-70B (NSQIP) | Absent | 81 | 0.900 | 1.000 | 0.947 |
|  | Present | 2 | 0.667 | 1.000 | 0.800 |
|  | Negated | 22 | 1.000 | 0.545 | 0.706 |
| LLaMA-3.2-3B (NSQIP) | Absent | 81 | 0.923 | 0.889 | 0.906 |
|  | Present | 2 | 0.500 | 0.500 | 0.500 |
|  | Negated | 22 | 0.680 | 0.773 | 0.723 |
| LLaMA-3.2-1B (NSQIP) | Absent | 81 | 0.852 | 0.926 | 0.888 |
|  | Present | 2 | 0.500 | 0.500 | 0.500 |
|  | Negated | 22 | 0.667 | 0.455 | 0.541 |

**Paresthesia**

| **Model (Dataset)** | **Class** | **n** | **Precision** | **Recall** | **F1-score** |
| --- | --- | --- | --- | --- | --- |
| LLaMA-3.3-70B (VNS) | Absent | 337 | 1.000 | 0.994 | 0.997 |
|  | Present | 8 | 0.778 | 0.875 | 0.824 |
|  | Negated | 17 | 0.889 | 0.941 | 0.914 |
| LLaMA-3.2-3B (VNS) | Absent | 337 | 0.982 | 0.961 | 0.972 |
|  | Present | 8 | 0.875 | 0.875 | 0.875 |
|  | Negated | 17 | 0.500 | 0.706 | 0.585 |
| LLaMA-3.2-1B (VNS) | Absent | 337 | 0.955 | 0.955 | 0.955 |
|  | Present | 8 | 0.250 | 0.750 | 0.375 |
|  | Negated | 17 | 1.000 | 0.059 | 0.111 |
| NSQIP | Not Evaluable | - | - | - | - |

**Excessive Sleep**

| **Model (Dataset)** | **Class** | **n** | **Precision** | **Recall** | **F1-score** |
| --- | --- | --- | --- | --- | --- |
| LLaMA-3.3-70B (VNS) | Absent | 337 | 0.997 | 0.991 | 0.994 |
|  | Present | 12 | 1.000 | 1.000 | 1.000 |
|  | Negated | 13 | 0.800 | 0.923 | 0.857 |
| LLaMA-3.2-3B (VNS) | Absent | 337 | 0.990 | 0.917 | 0.952 |
|  | Present | 12 | 0.917 | 0.917 | 0.917 |
|  | Negated | 13 | 0.289 | 0.846 | 0.431 |
| LLaMA-3.2-1B (VNS) | Absent | 337 | 0.929 | 0.964 | 0.946 |
|  | Present | 12 | 0.000 | 0.000 | 0.000 |
|  | Negated | 13 | 0.000 | 0.000 | 0.000 |
| NSQIP | Not Evaluable | - | - | - | - |
