# Supplementary material for: Longitudinal symptom severity tracking in vagus nerve stimulation patients: a 2-stage LLM-based pipeline with explainable AI
Source: JAMIA Open. 2026 Oct 1;9(5):ooag195. doi: 10.1093/jamiaopen/ooag195 (PMC13630440; doi:10.1093/jamiaopen/ooag195)
Supplement: Supplementary file 1 [file ooag195_supplementary_data.zip › Appendix SD.docx]

**Table S18.** Confusion Matrices by Symptom, Dataset, and Model. Each 3x3 matrix shows True (rows) vs. Predicted (columns) counts for Absent / Present / Negated.

**Seizure**

**LLaMA-3.3-70B -- VNS**

|  | **Absent** | **Present** | **Negated** |
| --- | --- | --- | --- |
| **Absent** | 257 | 0 | 2 |
| **Present** | 0 | 83 | 0 |
| **Negated** | 1 | 4 | 15 |

**LLaMA-3.3-70B -- NSQIP**

|  | **Absent** | **Present** | **Negated** |
| --- | --- | --- | --- |
| **Absent** | 80 | 0 | 0 |
| **Present** | 6 | 10 | 2 |
| **Negated** | 0 | 0 | 7 |

**LLaMA-3.2-3B -- VNS**

|  | **Absent** | **Present** | **Negated** |
| --- | --- | --- | --- |
| **Absent** | 247 | 8 | 4 |
| **Present** | 47 | 36 | 0 |
| **Negated** | 0 | 1 | 19 |

**LLaMA-3.2-3B -- NSQIP**

|  | **Absent** | **Present** | **Negated** |
| --- | --- | --- | --- |
| **Absent** | 78 | 2 | 0 |
| **Present** | 1 | 17 | 0 |
| **Negated** | 4 | 2 | 1 |

**LLaMA-3.2-1B -- VNS**

|  | **Absent** | **Present** | **Negated** |
| --- | --- | --- | --- |
| **Absent** | 247 | 3 | 9 |
| **Present** | 64 | 13 | 6 |
| **Negated** | 1 | 0 | 19 |

**LLaMA-3.2-1B -- NSQIP**

|  | **Absent** | **Present** | **Negated** |
| --- | --- | --- | --- |
| **Absent** | 78 | 2 | 0 |
| **Present** | 1 | 17 | 0 |
| **Negated** | 7 | 0 | 0 |

**Depression**

**LLaMA-3.3-70B -- VNS**

|  | **Absent** | **Present** | **Negated** |
| --- | --- | --- | --- |
| **Absent** | 203 | 2 | 1 |
| **Present** | 4 | 98 | 3 |
| **Negated** | 14 | 12 | 25 |

**LLaMA-3.3-70B -- NSQIP**

|  | **Absent** | **Present** | **Negated** |
| --- | --- | --- | --- |
| **Absent** | 53 | 1 | 3 |
| **Present** | 14 | 17 | 2 |
| **Negated** | 2 | 0 | 13 |

**LLaMA-3.2-3B -- VNS**

|  | **Absent** | **Present** | **Negated** |
| --- | --- | --- | --- |
| **Absent** | 177 | 28 | 1 |
| **Present** | 11 | 90 | 4 |
| **Negated** | 17 | 14 | 20 |

**LLaMA-3.2-3B -- NSQIP**

|  | **Absent** | **Present** | **Negated** |
| --- | --- | --- | --- |
| **Absent** | 50 | 1 | 6 |
| **Present** | 19 | 14 | 0 |
| **Negated** | 0 | 2 | 13 |

**LLaMA-3.2-1B -- VNS**

|  | **Absent** | **Present** | **Negated** |
| --- | --- | --- | --- |
| **Absent** | 198 | 2 | 6 |
| **Present** | 55 | 35 | 15 |
| **Negated** | 4 | 8 | 39 |

**LLaMA-3.2-1B -- NSQIP**

|  | **Absent** | **Present** | **Negated** |
| --- | --- | --- | --- |
| **Absent** | 54 | 0 | 3 |
| **Present** | 22 | 4 | 7 |
| **Negated** | 0 | 1 | 14 |

**Anxiety**

**LLaMA-3.3-70B -- VNS**

|  | **Absent** | **Present** | **Negated** |
| --- | --- | --- | --- |
| **Absent** | 235 | 0 | 0 |
| **Present** | 0 | 84 | 4 |
| **Negated** | 6 | 4 | 29 |

**LLaMA-3.3-70B -- NSQIP**

|  | **Absent** | **Present** | **Negated** |
| --- | --- | --- | --- |
| **Absent** | 58 | 8 | 1 |
| **Present** | 4 | 23 | 0 |
| **Negated** | 2 | 0 | 9 |

**LLaMA-3.2-3B -- VNS**

|  | **Absent** | **Present** | **Negated** |
| --- | --- | --- | --- |
| **Absent** | 221 | 14 | 0 |
| **Present** | 11 | 75 | 2 |
| **Negated** | 8 | 18 | 13 |

**LLaMA-3.2-3B -- NSQIP**

|  | **Absent** | **Present** | **Negated** |
| --- | --- | --- | --- |
| **Absent** | 62 | 0 | 5 |
| **Present** | 17 | 10 | 0 |
| **Negated** | 1 | 0 | 10 |

**LLaMA-3.2-1B -- VNS**

|  | **Absent** | **Present** | **Negated** |
| --- | --- | --- | --- |
| **Absent** | 217 | 12 | 6 |
| **Present** | 7 | 81 | 0 |
| **Negated** | 31 | 5 | 3 |

**LLaMA-3.2-1B -- NSQIP**

|  | **Absent** | **Present** | **Negated** |
| --- | --- | --- | --- |
| **Absent** | 62 | 3 | 2 |
| **Present** | 24 | 1 | 2 |
| **Negated** | 0 | 1 | 10 |

**Suicidal Ideation**

**LLaMA-3.3-70B -- VNS**

|  | **Absent** | **Present** | **Negated** |
| --- | --- | --- | --- |
| **Absent** | 264 | 0 | 2 |
| **Present** | 0 | 43 | 0 |
| **Negated** | 4 | 1 | 48 |

**LLaMA-3.3-70B -- NSQIP**

|  | **Absent** | **Present** | **Negated** |
| --- | --- | --- | --- |
| **Absent** | 85 | 0 | 0 |
| **Present** | 2 | 8 | 0 |
| **Negated** | 6 | 2 | 2 |

**LLaMA-3.2-3B -- VNS**

|  | **Absent** | **Present** | **Negated** |
| --- | --- | --- | --- |
| **Absent** | 263 | 0 | 3 |
| **Present** | 0 | 42 | 1 |
| **Negated** | 17 | 19 | 17 |

**LLaMA-3.2-3B -- NSQIP**

|  | **Absent** | **Present** | **Negated** |
| --- | --- | --- | --- |
| **Absent** | 85 | 0 | 0 |
| **Present** | 1 | 7 | 2 |
| **Negated** | 2 | 6 | 2 |

**LLaMA-3.2-1B -- VNS**

|  | **Absent** | **Present** | **Negated** |
| --- | --- | --- | --- |
| **Absent** | 262 | 3 | 1 |
| **Present** | 4 | 13 | 26 |
| **Negated** | 2 | 1 | 50 |

**LLaMA-3.2-1B -- NSQIP**

|  | **Absent** | **Present** | **Negated** |
| --- | --- | --- | --- |
| **Absent** | 85 | 0 | 0 |
| **Present** | 2 | 5 | 3 |
| **Negated** | 1 | 6 | 3 |

**Pain**

**LLaMA-3.3-70B -- VNS**

|  | **Absent** | **Present** | **Negated** |
| --- | --- | --- | --- |
| **Absent** | 246 | 21 | 0 |
| **Present** | 1 | 68 | 3 |
| **Negated** | 8 | 3 | 12 |

*LLaMA-3.3-70B -- NSQIP: NE (not evaluable)*

**LLaMA-3.2-3B -- VNS**

|  | **Absent** | **Present** | **Negated** |
| --- | --- | --- | --- |
| **Absent** | 235 | 1 | 31 |
| **Present** | 49 | 4 | 19 |
| **Negated** | 3 | 0 | 20 |

*LLaMA-3.2-3B -- NSQIP: NE (not evaluable)*

**LLaMA-3.2-1B -- VNS**

|  | **Absent** | **Present** | **Negated** |
| --- | --- | --- | --- |
| **Absent** | 249 | 0 | 18 |
| **Present** | 46 | 1 | 25 |
| **Negated** | 20 | 0 | 3 |

*LLaMA-3.2-1B -- NSQIP: NE (not evaluable)*

**Nausea and Vomiting**

**LLaMA-3.3-70B -- VNS**

|  | **Absent** | **Present** | **Negated** |
| --- | --- | --- | --- |
| **Absent** | 267 | 1 | 9 |
| **Present** | 1 | 15 | 0 |
| **Negated** | 24 | 0 | 45 |

**LLaMA-3.3-70B -- NSQIP**

|  | **Absent** | **Present** | **Negated** |
| --- | --- | --- | --- |
| **Absent** | 54 | 1 | 2 |
| **Present** | 0 | 12 | 1 |
| **Negated** | 13 | 3 | 19 |

**LLaMA-3.2-3B -- VNS**

|  | **Absent** | **Present** | **Negated** |
| --- | --- | --- | --- |
| **Absent** | 264 | 3 | 10 |
| **Present** | 0 | 15 | 1 |
| **Negated** | 41 | 0 | 28 |

**LLaMA-3.2-3B -- NSQIP**

|  | **Absent** | **Present** | **Negated** |
| --- | --- | --- | --- |
| **Absent** | 45 | 4 | 8 |
| **Present** | 2 | 10 | 1 |
| **Negated** | 21 | 2 | 12 |

**LLaMA-3.2-1B -- VNS**

|  | **Absent** | **Present** | **Negated** |
| --- | --- | --- | --- |
| **Absent** | 277 | 0 | 0 |
| **Present** | 3 | 3 | 10 |
| **Negated** | 32 | 2 | 35 |

**LLaMA-3.2-1B -- NSQIP**

|  | **Absent** | **Present** | **Negated** |
| --- | --- | --- | --- |
| **Absent** | 48 | 6 | 3 |
| **Present** | 1 | 9 | 3 |
| **Negated** | 22 | 3 | 10 |

**Fatigue**

**LLaMA-3.3-70B -- VNS**

|  | **Absent** | **Present** | **Negated** |
| --- | --- | --- | --- |
| **Absent** | 313 | 3 | 0 |
| **Present** | 21 | 12 | 0 |
| **Negated** | 1 | 0 | 12 |

*LLaMA-3.3-70B -- NSQIP: NE (not evaluable)*

**LLaMA-3.2-3B -- VNS**

|  | **Absent** | **Present** | **Negated** |
| --- | --- | --- | --- |
| **Absent** | 292 | 22 | 2 |
| **Present** | 30 | 3 | 0 |
| **Negated** | 1 | 0 | 12 |

*LLaMA-3.2-3B -- NSQIP: NE (not evaluable)*

**LLaMA-3.2-1B -- VNS**

|  | **Absent** | **Present** | **Negated** |
| --- | --- | --- | --- |
| **Absent** | 295 | 1 | 20 |
| **Present** | 31 | 2 | 0 |
| **Negated** | 0 | 2 | 11 |

*LLaMA-3.2-1B -- NSQIP: NE (not evaluable)*

**Self-harm**

**LLaMA-3.3-70B -- VNS**

|  | **Absent** | **Present** | **Negated** |
| --- | --- | --- | --- |
| **Absent** | 317 | 0 | 0 |
| **Present** | 0 | 2 | 0 |
| **Negated** | 0 | 1 | 42 |

**LLaMA-3.3-70B -- NSQIP**

|  | **Absent** | **Present** | **Negated** |
| --- | --- | --- | --- |
| **Absent** | 93 | 0 | 0 |
| **Present** | 0 | 4 | 0 |
| **Negated** | 1 | 1 | 6 |

**LLaMA-3.2-3B -- VNS**

|  | **Absent** | **Present** | **Negated** |
| --- | --- | --- | --- |
| **Absent** | 307 | 1 | 9 |
| **Present** | 1 | 1 | 0 |
| **Negated** | 3 | 0 | 40 |

**LLaMA-3.2-3B -- NSQIP**

|  | **Absent** | **Present** | **Negated** |
| --- | --- | --- | --- |
| **Absent** | 92 | 1 | 0 |
| **Present** | 1 | 3 | 0 |
| **Negated** | 1 | 0 | 7 |

**LLaMA-3.2-1B -- VNS**

|  | **Absent** | **Present** | **Negated** |
| --- | --- | --- | --- |
| **Absent** | 305 | 1 | 11 |
| **Present** | 1 | 1 | 0 |
| **Negated** | 5 | 0 | 38 |

**LLaMA-3.2-1B -- NSQIP**

|  | **Absent** | **Present** | **Negated** |
| --- | --- | --- | --- |
| **Absent** | 92 | 0 | 1 |
| **Present** | 0 | 3 | 1 |
| **Negated** | 2 | 0 | 6 |

**Headaches**

**LLaMA-3.3-70B -- VNS**

|  | **Absent** | **Present** | **Negated** |
| --- | --- | --- | --- |
| **Absent** | 286 | 1 | 4 |
| **Present** | 0 | 41 | 0 |
| **Negated** | 6 | 3 | 21 |

**LLaMA-3.3-70B -- NSQIP**

|  | **Absent** | **Present** | **Negated** |
| --- | --- | --- | --- |
| **Absent** | 49 | 0 | 1 |
| **Present** | 8 | 21 | 4 |
| **Negated** | 0 | 1 | 21 |

**LLaMA-3.2-3B -- VNS**

|  | **Absent** | **Present** | **Negated** |
| --- | --- | --- | --- |
| **Absent** | 263 | 26 | 2 |
| **Present** | 13 | 28 | 0 |
| **Negated** | 3 | 0 | 27 |

**LLaMA-3.2-3B -- NSQIP**

|  | **Absent** | **Present** | **Negated** |
| --- | --- | --- | --- |
| **Absent** | 49 | 1 | 0 |
| **Present** | 0 | 30 | 3 |
| **Negated** | 13 | 5 | 4 |

**LLaMA-3.2-1B -- VNS**

|  | **Absent** | **Present** | **Negated** |
| --- | --- | --- | --- |
| **Absent** | 284 | 1 | 6 |
| **Present** | 29 | 2 | 10 |
| **Negated** | 1 | 0 | 29 |

**LLaMA-3.2-1B -- NSQIP**

|  | **Absent** | **Present** | **Negated** |
| --- | --- | --- | --- |
| **Absent** | 50 | 0 | 0 |
| **Present** | 15 | 10 | 8 |
| **Negated** | 0 | 0 | 22 |

**Gastroesophageal Reflux (GERD)**

**LLaMA-3.3-70B -- VNS**

|  | **Absent** | **Present** | **Negated** |
| --- | --- | --- | --- |
| **Absent** | 328 | 0 | 6 |
| **Present** | 1 | 10 | 0 |
| **Negated** | 1 | 0 | 16 |

**LLaMA-3.3-70B -- NSQIP**

|  | **Absent** | **Present** | **Negated** |
| --- | --- | --- | --- |
| **Absent** | 68 | 0 | 3 |
| **Present** | 2 | 12 | 0 |
| **Negated** | 1 | 0 | 19 |

**LLaMA-3.2-3B -- VNS**

|  | **Absent** | **Present** | **Negated** |
| --- | --- | --- | --- |
| **Absent** | 326 | 1 | 7 |
| **Present** | 1 | 10 | 0 |
| **Negated** | 9 | 0 | 8 |

**LLaMA-3.2-3B -- NSQIP**

|  | **Absent** | **Present** | **Negated** |
| --- | --- | --- | --- |
| **Absent** | 70 | 1 | 0 |
| **Present** | 1 | 13 | 0 |
| **Negated** | 12 | 5 | 3 |

**LLaMA-3.2-1B -- VNS**

|  | **Absent** | **Present** | **Negated** |
| --- | --- | --- | --- |
| **Absent** | 331 | 0 | 3 |
| **Present** | 1 | 10 | 0 |
| **Negated** | 16 | 0 | 1 |

**LLaMA-3.2-1B -- NSQIP**

|  | **Absent** | **Present** | **Negated** |
| --- | --- | --- | --- |
| **Absent** | 70 | 1 | 0 |
| **Present** | 2 | 12 | 0 |
| **Negated** | 17 | 3 | 0 |

**Rash**

**LLaMA-3.3-70B -- VNS**

|  | **Absent** | **Present** | **Negated** |
| --- | --- | --- | --- |
| **Absent** | 243 | 0 | 18 |
| **Present** | 0 | 5 | 1 |
| **Negated** | 7 | 0 | 88 |

**LLaMA-3.3-70B -- NSQIP**

|  | **Absent** | **Present** | **Negated** |
| --- | --- | --- | --- |
| **Absent** | 81 | 0 | 0 |
| **Present** | 0 | 2 | 0 |
| **Negated** | 9 | 1 | 12 |

**LLaMA-3.2-3B -- VNS**

|  | **Absent** | **Present** | **Negated** |
| --- | --- | --- | --- |
| **Absent** | 223 | 1 | 37 |
| **Present** | 1 | 5 | 0 |
| **Negated** | 23 | 0 | 72 |

**LLaMA-3.2-3B -- NSQIP**

|  | **Absent** | **Present** | **Negated** |
| --- | --- | --- | --- |
| **Absent** | 72 | 1 | 8 |
| **Present** | 1 | 1 | 0 |
| **Negated** | 5 | 0 | 17 |

**LLaMA-3.2-1B -- VNS**

|  | **Absent** | **Present** | **Negated** |
| --- | --- | --- | --- |
| **Absent** | 251 | 3 | 7 |
| **Present** | 0 | 5 | 1 |
| **Negated** | 49 | 1 | 45 |

**LLaMA-3.2-1B -- NSQIP**

|  | **Absent** | **Present** | **Negated** |
| --- | --- | --- | --- |
| **Absent** | 75 | 1 | 5 |
| **Present** | 1 | 1 | 0 |
| **Negated** | 12 | 0 | 10 |

**Paresthesia**

**LLaMA-3.3-70B -- VNS**

|  | **Absent** | **Present** | **Negated** |
| --- | --- | --- | --- |
| **Absent** | 335 | 1 | 1 |
| **Present** | 0 | 7 | 1 |
| **Negated** | 0 | 1 | 16 |

*LLaMA-3.3-70B -- NSQIP: NE (not evaluable)*

**LLaMA-3.2-3B -- VNS**

|  | **Absent** | **Present** | **Negated** |
| --- | --- | --- | --- |
| **Absent** | 324 | 1 | 12 |
| **Present** | 1 | 7 | 0 |
| **Negated** | 5 | 0 | 12 |

*LLaMA-3.2-3B -- NSQIP: NE (not evaluable)*

**LLaMA-3.2-1B -- VNS**

|  | **Absent** | **Present** | **Negated** |
| --- | --- | --- | --- |
| **Absent** | 322 | 15 | 0 |
| **Present** | 2 | 6 | 0 |
| **Negated** | 13 | 3 | 1 |

*LLaMA-3.2-1B -- NSQIP: NE (not evaluable)*

**Excessive Sleep**

**LLaMA-3.3-70B -- VNS**

|  | **Absent** | **Present** | **Negated** |
| --- | --- | --- | --- |
| **Absent** | 334 | 0 | 3 |
| **Present** | 0 | 12 | 0 |
| **Negated** | 1 | 0 | 12 |

*LLaMA-3.3-70B -- NSQIP: NE (not evaluable)*

**LLaMA-3.2-3B -- VNS**

|  | **Absent** | **Present** | **Negated** |
| --- | --- | --- | --- |
| **Absent** | 309 | 1 | 27 |
| **Present** | 1 | 11 | 0 |
| **Negated** | 2 | 0 | 11 |

*LLaMA-3.2-3B -- NSQIP: NE (not evaluable)*

**LLaMA-3.2-1B -- VNS**

|  | **Absent** | **Present** | **Negated** |
| --- | --- | --- | --- |
| **Absent** | 325 | 10 | 2 |
| **Present** | 12 | 0 | 0 |
| **Negated** | 13 | 0 | 0 |

*LLaMA-3.2-1B -- NSQIP: NE (not evaluable)*
